# Synergistic delivery of ginseng exosomes and biomimetic melanosomes via temporally controlled hydrogel microneedles restrain the pathologic triad of vitiligo

**DOI:** 10.1186/s12951-026-04168-w

**Published:** 2026-03-02

**Authors:** Luyue Chang, Junqi Xiang, Ting Zhang, Yanna Ban, Lihua Kang, Yujuan Wu, Li Du, Shasha Zhu, Yao Gong, Xiaoying Zhang, Li Wang, Jin Chen, Wei Cheng, Jie Xu

**Affiliations:** 1 The Center for Clinical Molecular Medical Detection, Innovative and Translational Laboratory of Molecular Diagnostics, Laboratory Medicine Center, The First Affiliated Hospital of Chongqing Medical University, Chongqing, 400016 China; 2 Department of Dermatology, The First Affiliated Hospital of Chongqing Medical University, Chongqing, 400016 China; 3 College of Basic Medicine, Chongqing Medical University, Chongqing, 400016 China; 4 Biobank, The First Affiliated Hospital of Chongqing Medical University, Chongqing, 400016 China; 5 Reproductive Medicine Center, The First Affiliated Hospital of Chongqing Medical University, Chongqing, 400016 China; 6 Department of Clinical Laboratory, Chongqing Health Center for Women and Children’s Hospital of Chongqing Medical University, Chongqing, 401147 China

**Keywords:** Vitiligo, Ginseng-derived exosomes, Hydrogel microneedles, Oxidative stress, Repigmentation

## Abstract

Vitiligo pathogenesis involves progressive melanocyte loss and keratinocyte dysfunction, which are driven primarily by oxidative stress resulting from excessive ROS accumulation. We engineered a temporally controlled hydrogel microneedle system that integrates ginseng-derived exosomes (G-Exos) with biomimetic polydopamine nanoparticles (PDA@PEGs) to concurrently target the pathogenic triad of vitiligo, including oxidative stress, inflammation, and melanocyte deficiency. This system employs methacrylated hyaluronic acid (HAMA) hydrogel microneedles for rapid PDA@PEG release while utilizing glyceryl monostearate micelles to achieve matrix metalloproteinase-9 (MMP-9)-responsive G-Exo release at inflammatory foci, enabling intelligent spatiotemporal control. Functionally, G-Exos help restore redox homeostasis and suppress inflammation through bioactive constituents, thereby protecting melanocytes and enhancing keratinocyte proliferation. Moreover, PDA@PEG promotes repigmentation through the dual mechanisms of exogenous melanin deposition and endogenous melanogenesis stimulation. In murine models, this strategy achieves significant repigmentation within 3 weeks by activating follicular stem cells, upregulating melanogenic markers (Tyr/Mc1r), increasing antioxidant defense (ApoE), and suppressing inflammatory signaling (IL-17). This natural-biomimetic hybrid design leverages biocompatible materials to co-target multiple pathological axes, offering a novel self-adaptive approach for microenvironmental rehabilitation in vitiligo.

**Graphical Abstract:**

**Figure:**
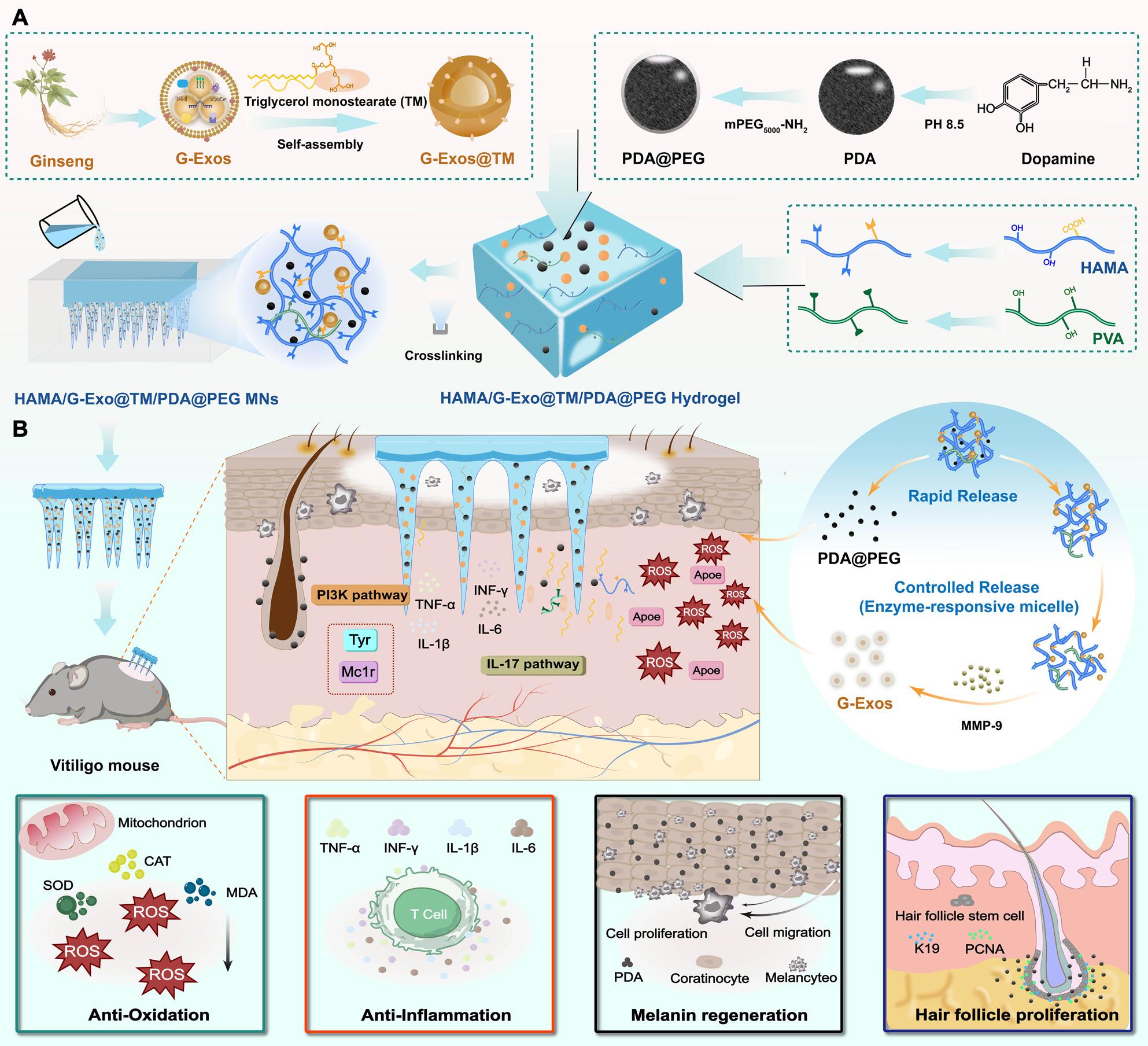
Schematic illustration of the temporally controlled hydrogel MN patch for vitiligo treatment. (A) Fabrication process of temporally controlled hydrogel MNs. (B) Therapeutic mechanism: MNs achieve rapid and lesion-targeted repigmentation through spatiotemporally programmed delivery of G-Exos and PDA@PEG, which synergistically address the core pathological features of vitiligo: oxidative stress, the inflammatory response, and melanocyte dysfunction.

**Supplementary Information:**

The online version contains supplementary material available at 10.1186/s12951-026-04168-w.

## Introduction

Vitiligo is a chronic depigmentary disorder characterized by the progressive loss of melanocytes and keratinocyte dysfunction, leading to impaired melanin synthesis and transport, with a global prevalence of approximately 1%-2% [1, 2]. The disease predominantly affects sun-exposed areas such as the neck and limbs, not only causing psychological distress but also significantly increasing skin cancer risk due to the compromised photoprotection capacity of the epidermis [3]. Currently, the precise etiology and pathogenesis of vitiligo are incompletely understood, but the core pathophysiology revolves around a self-perpetuating “pathologic triad”: abnormal oxidative stress initiating melanocyte damage, ensuing immuno-inflammatory cascades amplifying destruction, and resulting in melanocyte loss/dysfunction, causing refractory depigmentation. Central to this triad is oxidative stress. Melanocytes in vitiligo patients exhibit intrinsic defects that increase their sensitivity to extracellular stimuli, resulting in excessive ROS accumulation [4]. Patients exhibit dysregulated expression of oxidative-antioxidant genes and disrupted redox homeostasis, with impaired expression of multiple antioxidant enzymes, such as catalase (CAT), superoxide dismutase (SOD), and glutathione peroxidase (GPx) [5]. Sustained oxidative stress further triggers an immuno-inflammatory cascade by promoting T lymphocyte activation and inflammatory cytokine release [6], enhancing melanocyte immunogenicity, and inducing keratinocyte secretion of chemokines that recruit cytotoxic T cells [7]. This establishes a self-perpetuating cycle of progressive melanocyte destruction. Confronting this pathologic triad necessitates integrated therapeutic strategies targeting oxidative stress, inflammation, and melanocyte dysfunction.

Unfortunately, current mainstream therapies, while effective in controlling progression, often address only isolated axes of this triad and have significant limitations in achieving stable repigmentation. Topical calcineurin inhibitors suppress T-cell activity and inflammation but exhibit limited efficacy outside facial regions, cause local irritation and do not fully reverse underlying melanocyte dysfunction [8]. Narrowband ultraviolet B (NB-UVB) phototherapy activates melanocyte stem cells but is hampered by prolonged duration, low complete repigmentation rates, and high recurrence within a year post-treatment [9]. Surgical transplantation offers repigmentation for stable cases but is restricted by the Koebner phenomenon, the risk of trauma, and uneven results [10, 11]. These limitations underscore the need for novel approaches capable of synchronously modulating the interconnected pathologic axes.

To address the crucial need for microenvironmental rehabilitation, plant-derived nanovesicles (pNVs) offer a compelling approach because of their innate biocompatibility, biodegradability [12, 13], and enhanced cellular uptake via their biomimetic structure [14]. pNVs can independently deliver bioactive compounds to exert multifaceted therapeutic effects [15], including redox homeostasis restoration (e.g., by scavenging excess epidermal H₂O₂) [16] and immunomodulation [17]. Consequently, leveraging the renowned antioxidant and anti-inflammatory potency of Panax ginseng [18–20], its derived exosomes (G-Exos) have emerged as ideal candidates for restoring melanocyte functionality and immune homeostasis. G-Exos potentially overcome the limitations of synthetic drugs through their natural origin and inherent stability, reducing systemic exposure risks while promoting a conducive microenvironment for repigmentation. However, microenvironmental restoration alone is insufficient to achieve complete cosmetic recovery; effective repigmentation also necessitates direct pigment replenishment. In this context, exogenous melanin supplementation represents a promising strategy to address the depigmentation axis. Since sourcing human melanin is impractical, biomimetic alternatives such as polydopamine nanoparticles (PDA) have been developed [21]. Inspired by melanosomes, the structural properties of PDA enable efficient endocytic transport [22, 23]. In addition to providing immediate optical compensation through pigment deposition, PDA potently scavenges ROS via its phenolic hydroxyl groups [24], thereby synergistically enhancing antioxidant defense. While individual properties of G-Exos and PDA are known, a unified strategy that coordinates their complementary actions is lacking. Existing approaches do not provide the spatiotemporal control needed to address vitiligo’s pathological triad, creating a need for a platform that enables sequential and targeted delivery.

**Scheme 1:**
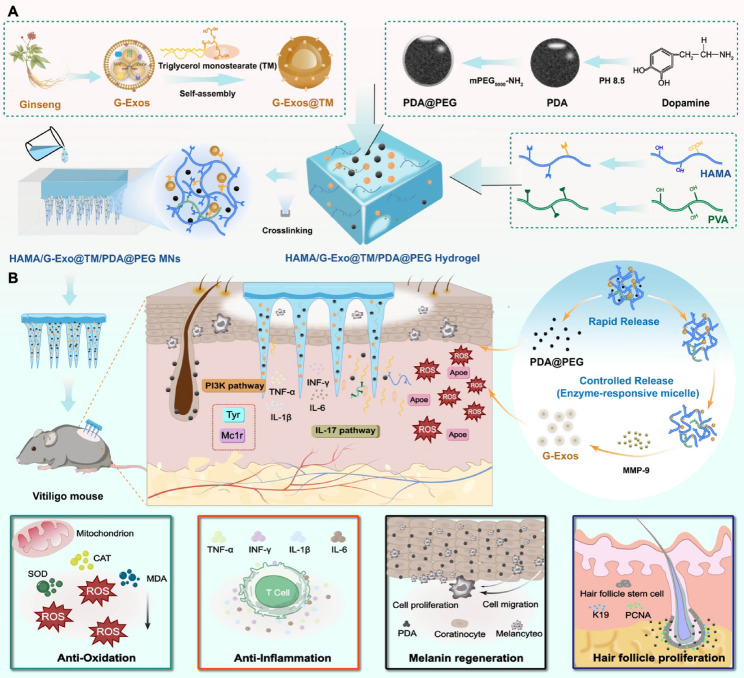
Schematic illustration of the temporally controlled hydrogel MN patch for vitiligo treatment. (**A**) Fabrication process of temporally controlled hydrogel MNs. (**B**) Therapeutic mechanism: MNs achieve rapid and lesion-targeted repigmentation through spatiotemporally programmed delivery of G-Exos and PDA@PEG, which synergistically address the core pathological features of vitiligo: oxidative stress, the inflammatory response, and melanocyte dysfunction

In this study, we developed “Temporally Controlled Hydrogel Microneedles (MNs)” via a nature-biomimetic hybrid design to target vitiligo’s pathologic triad of oxidative stress, inflammation, and melanocyte dysfunction, aiming to promote rapid lesion-targeted repigmentation. Central to this strategy are G-Exos, which help restore redox homeostasis, suppress inflammatory cascades, protect melanocytes from oxidative damage, and support keratinocyte proliferation to improve the melanocyte niche, and PDA@PEG, which utilizes PEG-enhanced stability to deliver exogenous pigment while stimulating endogenous melanogenesis. To ensure spatiotemporally controlled delivery of this therapeutic combination to vitiliginous lesions, we engineered an intelligent MN platform that responds to the pathological overexpression of matrix metalloproteinases (MMPs), particularly MMP-9, within these areas [25, 26]. Using triglycerol monostearate (TM) to form enzyme-responsive micelles [27, 28] and a HAMA/PVA hydrogel for the matrix, we fabricated MNs designed for a biphasic release profile. This composite structure was selected to provide mechanical properties suitable for skin insertion and to support different release pathways for the two cargos. Upon skin penetration and dissolution, the PDA@PEG nanoparticles coloaded within the dissolving hydrogel matrix are rapidly released to prompt immediate pigment deposition and antioxidant action at the lesion site. Simultaneously, the released micellar carriers undergo site-specific disassembly in the elevated MMP-9 microenvironment, enabling on-demand, lesion-confined and sustained release of encapsulated G-Exos to initiate microenvironmental reprogramming. This spatiotemporally orchestrated process—rapid PDA@PEG release coupled with sustained, on-demand G-Exo release activated by the MMP-9-rich microenvironment—synergistically promoted repigmentation by enhancing antioxidant, anti-inflammatory, and pro-melanogenic activities (Scheme 1). By concurrently addressing the core pathologic triad of vitiligo through temporally controlled delivery, this multifunctional, lesion-targeted strategy offers a novel, integrated approach to mitigating existing therapeutic limitations and provides a basis for future translational development.

## Results and discussion

### Isolation, compositional profiling, and cytoprotective function of G-Exos

**Fig. 1:**
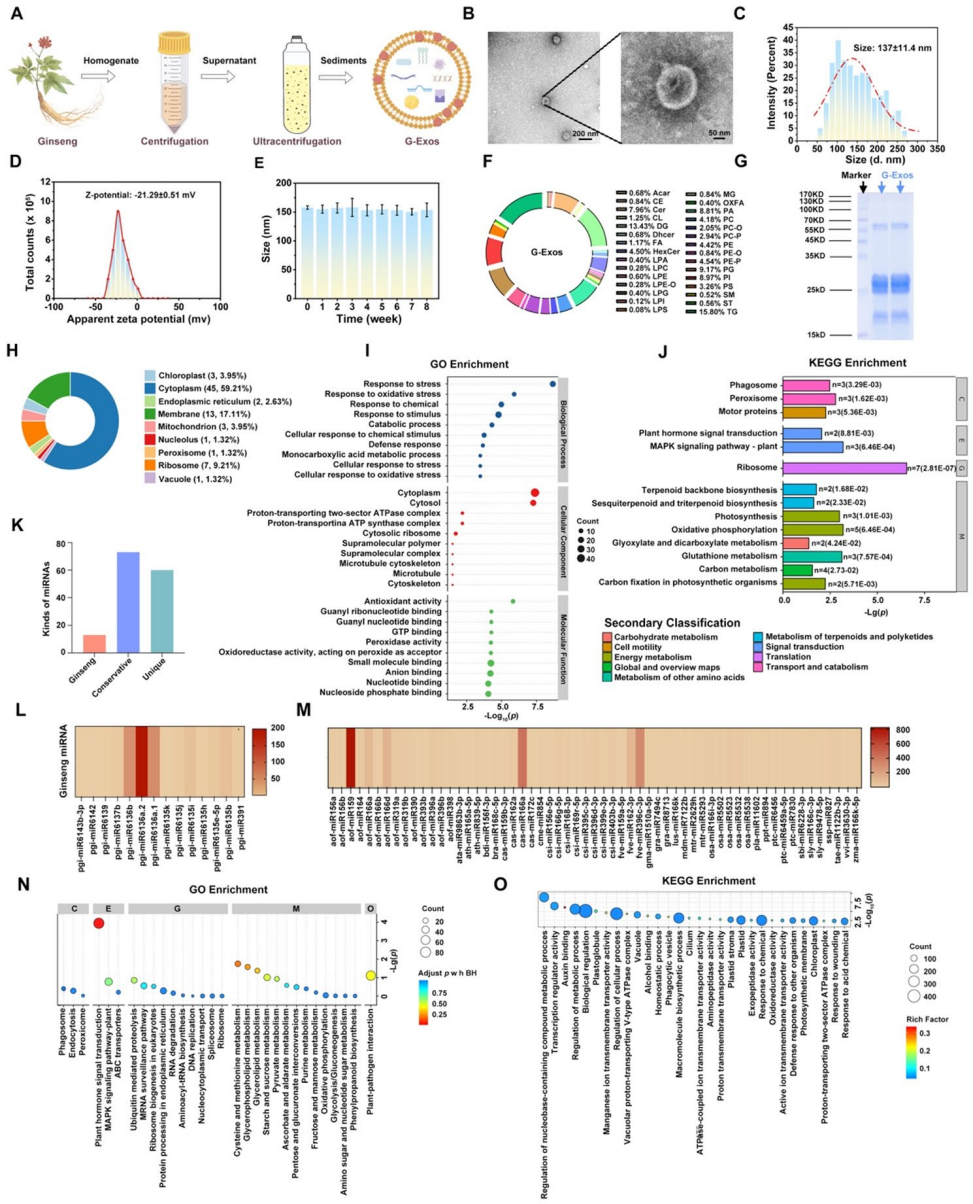
Isolation, compositional profiling, and cytoprotective function of G-Exos. (**A**) Schematic illustration of G-Exos isolation. (**B**) TEM image of G-Exos collected from the 30/45% interface of a sucrose density gradient. (**C-D**) Particle size distribution (**C**) and zeta potential (**D**) of G-Exos. (**E**) Stability assessment of G-Exos stored at − 80 °C over time. (**F**) Relative quantification of lipid classes in G-Exos. (**G**) Protein profile of G-Exos separated by SDS‒PAGE and visualized with Coomassie Brilliant Blue staining. (**H**) Subcellular localization prediction of identified G-Exo proteins. (**I**) GO functional annotation of G-Exo proteins. (**J**) KEGG pathway enrichment analysis of the top 30 abundant proteins in G-Exos. (**K**) Frequencies and types of conserved miRNAs (excluding miRNAs that match the ginseng genome), unique miRNAs and miRNAs that are mapped to the ginseng genome database. (**L**) Heatmap of miRNA expression profiles aligned to the ginseng genome database. (**M**) Heatmap of conserved miRNAs that did not align to the ginseng genome. (**N**) GO term enrichment analysis of the predicted miRNA target genes. (**O**) KEGG pathway enrichment analysis of miRNA target genes. Data are presented as the means ± SD: *p < 0.05, **p < 0.01, ***p < 0.001

The isolation and purification of G-Exos from root juice were achieved via differential centrifugation and sucrose density gradient ultracentrifugation (Fig. 1A). The yield was 50 mg of G-Exos protein/kg fresh root. In line with the evolving perspective in the field, which emphasizes that adequately enriched EVs obtained by well-controlled isolation methods may hold greater practical value for functional applications than the pursuit of absolute purity [29], our protocol prioritized a balance between integrity, yield, and enrichment. TEM analysis revealed a characteristic cup-shaped morphology with homogeneous dispersion (Fig. 1B). The particle size distribution measured by dynamic light scattering showed a peak at 137.1 ± 11.4 nm (Fig. 1C), with a zeta potential of − 21.29 ± 0.51 mV, indicating colloidal stability (Fig. 1D). The G-Exos retained structural integrity for ≥ 2 months at − 80 °C (Fig. 1E).

Plant-derived exosomes selectively encapsulate bioactive molecules from their parent cells [30]. To elucidate their potential in modulating skin pathophysiology, we systematically characterized G-Exo components. Lipidomic analysis revealed 2,487 lipid species across 30 subclasses in G-Exos (Fig. S1). Notably, triglycerides (TGs, 15.8%) and diacylglycerols (DGs, 13.4%) were predominant (Fig. 1F). As key lipid mediators, TG/DG modulates membrane dynamics and cellular signaling, which are crucial for regulating oxidative stress responses and intercellular communication. Proteomic profiling revealed 93 proteins in G-Exos (Fig. S2A). The major protein bands were located in the 15–25, 25, and 55–70 kDa regions (Fig. 1G). Subcellular localization analysis revealed a predominant cytoplasmic origin, with minor chloroplast/ER/membrane associations (Fig. 1H). GO enrichment confirmed strong links to catalytic activity and antioxidant processes, including peroxidase activity and the oxidative stress response (Fig. 1I). KEGG analysis further revealed enrichment of glutathione metabolism and oxidative phosphorylation core pathways counteracting ROS-mediated pigment cell damage (Fig. 1J). Importantly, these genes are also related to proteins closely linked to the MAPK signaling pathway. The protein interaction network shown in Fig. S2 and Table S1 revealed close and extensive interactions between the proteins identified. Small RNA sequencing identified 73 miRNAs (18–36 nt), with the top 20 species constituting 93.88% of the total miRNA expression (Fig. S3A-C). BLAST analysis against miRBase v22.0 confirmed their conserved nature, revealing 13 perfect matches to the ginseng genome (Fig. 1L) and 60 novel sequences containing unique 3p- and 5p-arm derivatives (Fig. 1K, M). The analyses included Rfam classification statistics, predicted precursor secondary structures, and nucleotide preference profiles (Fig. S3D, S4-5). These findings support emerging evidence that plant exosomal miRNAs may regulate mammalian cells through cross-species RNA interference [31]. GO analysis predicted miRNA targets involved in peroxisome function, MAPK signaling, and oxidative phosphorylation (Fig. 1N), and KEGG enrichment further implicated oxidoreductase activity-related pathways (Fig. 1O). These findings suggest that G-Exo miRNAs potentially modulate oxidative stress and inflammatory responses in mammalian systems.

Given the ROS-driven melanocyte apoptosis and inflammatory cytokine milieu characteristic of vitiligo lesions, we evaluated the ability of G-Exos to restore redox homeostasis in H₂O₂-induced oxidative stress models (0.8 µmol/mL, Fig. S6). At a concentration of 10 µg/mL, G-Exos reversed oxidative stress-induced death in human melanocytes (PIG1) while increasing the proliferation of keratinocytes (HaCaT) across the therapeutic concentration range (0–100 µg/mL; Fig. S7) in a dose-dependent manner. This is a critical finding given the keratinocyte‒melanocyte crosstalk in epidermal pigmentation [32]. The treatment demonstrated concentration-dependent therapeutic effects, progressively restoring antioxidant defenses through significant increases in SOD and CAT (*p* < 0.001) activities (Fig. S8A-B). Lipid peroxidation was incrementally reduced, with a maximal 74.3% decrease in MDA content achieved at 100 µg/mL (*p* < 0.001, Fig. S8C). Furthermore, compared with no treatment, G-Exos concentration-dependently suppressed the production of proinflammatory cytokines, resulting in significant reductions in both TNF-α and IL-6 secretion (*p* < 0.001, Fig. S9). These results align with the literature on the therapeutic potential of EVs in skin disorders [33], highlighting the promise of G-Exos as a targeted treatment for vitiligo by restoring redox homeostasis and suppressing inflammation.

### Physicochemical characterization and cytoprotective effects of PDA@PEG

**Fig. 2:**
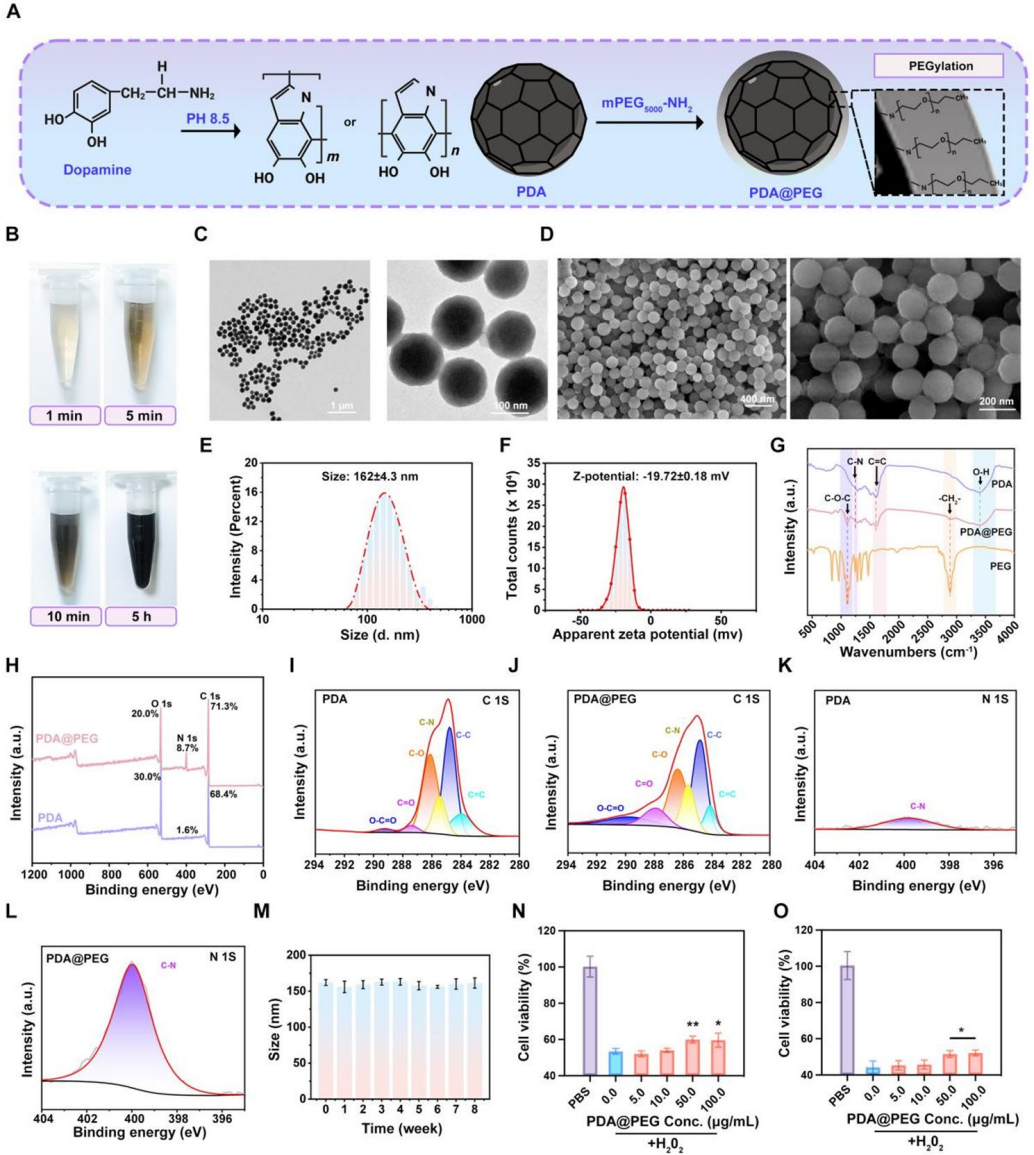
Physicochemical characterization and cytoprotective effect of PDA@PEG. (**A**) Schematic illustration of the structure of PDA@PEG. (**B**) Temporal colorimetric evolution of the PDA@PEG reaction system. (**C-D**) Representative TEM (**C**) and SEM (**D**) images of PDA@PEG. (**E-F**) Size distribution (**E**) and zeta potential (**F**) of PDA@PEG. (**G**) FT-IR spectra of PDA, PEG, and PDA@PEG. (**H**) XPS survey spectra of PDA and PDA@PEG. (**I-J**) High-resolution C 1s spectra of PDA (**I**) and PDA@PEG (**J**). (**K-L**) High-resolution N 1s spectra of PDA (**K**) and PDA@PEG (**L**). (**M**) Stability evaluation of PDA@PEG stored at 25 °C for 8 weeks. (**N-O**) Viability of HaCaT (**N**) and P1G1 (**O**) cells treated with PBS or different concentrations of PDA@PEG for 24 h following exposure to 0.8 µmol/mL H₂O₂. Data are presented as the means ± SD: *p < 0.05, **p < 0.01, ***p < 0.001

Polyethylene glycol-modified polydopamine (PDA@PEG) nanoparticles were synthesized via the oxidative polymerization of dopamine under alkaline conditions following a modified literature protocol [22, 24]. PEG modification was strategically employed to leverage its hydrophilic ethylene glycol repeat units for spatial stabilization: by reducing charge-based interactions between proteins/small molecules and nanoparticles, PEG effectively minimizes the adsorption of cutaneous secretory proteins onto NP surfaces, thereby increasing colloidal stability while increasing solubility in biological media and reducing cytotoxicity [34]. Covalent conjugation of mPEG5000-NH₂ to the PDA core was achieved through Schiff base/Michael addition chemistry (Fig. 2A) [35], a modification aimed at enhancing physiological stability. Successful PDA formation was confirmed by the characteristic color transition from pale yellow to opaque black during synthesis (Fig. 2B). The resulting nanoparticles retained the intrinsic catechol/quinone redox moieties of PDA, structures known to confer ROS scavenging capacity via electron donation [36]. Transmission and scanning electron microscopy (TEM/SEM) characterization revealed a monodisperse spherical morphology with an average diameter of 162 ± 4.3 nm (Fig. 2C-E). Colloidal stability was verified by zeta potential measurements (-19.72 ± 0.18 mV, Fig. 2F). Fourier transform infrared (FT-IR) spectroscopy confirmed successful PEGylation, revealing characteristic PDA peaks (3401 cm⁻¹ for O-H stretch; 1604 cm⁻¹ for aromatic C = C) alongside new PEG signatures (2879 cm⁻¹ for -CH₂- stretch; 1104 cm⁻¹ for C-O-C; and 1289 cm⁻¹ for C-N covalent linkage) (Fig. 2G) [37, 38]. X-ray photoelectron spectroscopy (XPS) further validated surface functionalization (Fig. 2H-L), which notably increased the C/N atomic ratios and decreased the O content, with the C 1s/N 1s fine spectra revealing increased C‒N peaks. The PDA@PEG nanoparticles maintained excellent stability for 60 days at 25 °C (Fig. 2M) and showed concentration-dependent cytoprotective effects in oxidative stress models in H₂O₂-treated HaCaT and PIG1 cells. Treatment achieved maximal proliferation enhancement at 50 µg/mL, which was subsequently adopted as the concentration for all in vitro experiments. (Fig. 2N-O).

### Antioxidant, anti-inflammatory, and melanin-upregulating effects of PDA@PEG and G-Exos

**Fig. 3:**
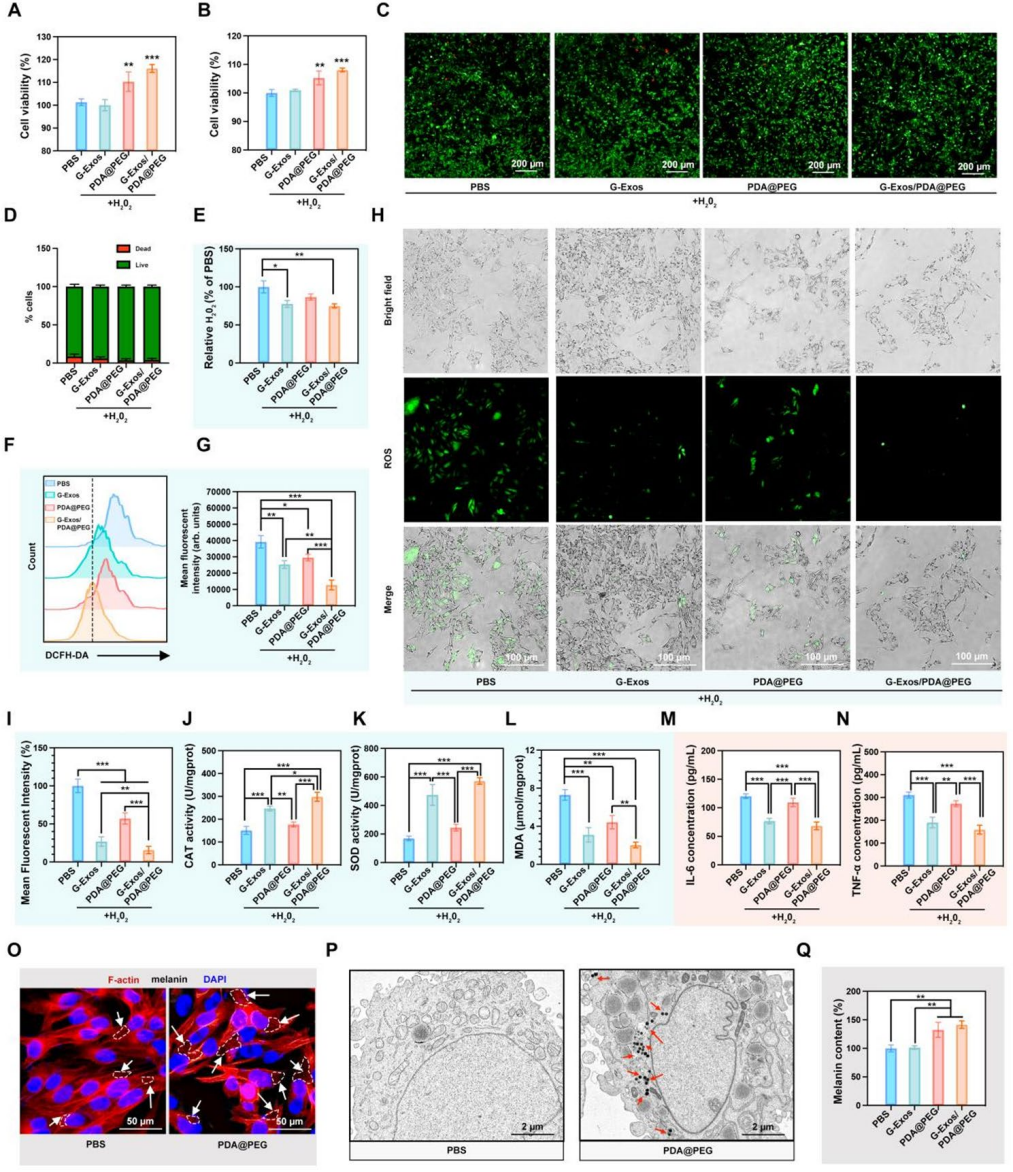
Antioxidant, anti-inflammatory, and melanin-upregulating effects of PDA@PEG and G-Exos. (**A–B**) Viability of HaCaT cells (**A**) and PIG1 cells (**B**) after 24 h of treatment with 0.8 µmol/mL H₂O₂. (**C-D**) Live/dead staining (**C**) and quantitative viability (**D**) of HaCaT cells after treatment. (**E**) Residual H₂O₂ concentration in the culture media after the interventions. (**F-G**) Flow cytometric analysis (**F**) and quantification (**G**) of ROS levels in treated cells. (**H–I**) Mitochondrial ROS levels in PIG1 cells challenged with 0.8 µmol/mL H₂O₂, as assessed by DCFH-DA staining (**H**) and quantitative analysis (**I**). (**J-L**) Antioxidant biomarkers in HaCaT cells: (**J**) CAT activity, (**K**) SOD activity, (**L**) MDA content. (**M-N**) Cytokine secretion in LPS-stimulated HaCaT cells: (**M**) IL-6, (**N**) TNF-α. (**O**) Cellular internalization of PDA@PEG in P1G1 cells. Melanosomes are indicated by white arrows. (**P**) Bio-TEM images of P1G1 cells after 24 h of co-incubation with PDA@PEG; melanosomes are marked by red arrows. (**Q**) Melanin synthesis rate in PIG1 cells across treatments. Data are presented as the means ± SD: *p < 0.05, **p < 0.01, ***p < 0.001

Having established the individual cytoprotective properties of G-Exos and PDA@PEG, we next sought to investigate their combined effect. The combination of G-Exos and PDA@PEG significantly reversed H₂O₂-induced cytotoxicity in both HaCaT keratinocytes and PIG1 melanocytes, as measured by a CCK-8 assay (Fig. 3A-B). Live/dead staining further revealed that, compared with other conditions, PDA@PEG provided superior cytoprotection (Fig. 3C-D). G-Exos exclusively eliminated extracellular H₂O₂ (*p* < 0.05), whereas PDA@PEG alone had no significant effect on scavenging capacity. Notably, the G-Exo/PDA@PEG combination further enhanced H₂O₂ clearance (*p* < 0.01), demonstrating synergistic scavenging activity (Fig. 3E). DCFH-DA flow cytometry revealed that G-Exos were more potent scavengers than PDA@PEGs were, a hierarchy corroborated by fluorescence imaging showing maximal signal attenuation in the combination group (Fig. 3F-I). In addition to radical scavenging, the G-Exos significantly restored SOD and CAT activity (*p* < 0.01 vs. PDA@PEG) while also markedly reducing the MDA level (*p* < 0.001), and these effects were further amplified in the presence of PDA@PEG (Fig. 3J-L). This antioxidant synergy paralleled the anti-inflammatory response, where G-Exos alone suppressed TNF-α and IL-6 secretion in tumors by 62% and 57%, respectively, and this effect was potentiated by combination therapy (Fig. 3M-N). The observed synergy likely originates from the complementary mechanisms of the two agents: G-Exos reprogram the inflammatory microenvironment and directly enhance cellular antioxidant defenses, thereby creating a more receptive cellular state for the subsequent pigment-inducing and ROS-scavenging actions of PDA@PEG. To confirm the cellular internalization of PDA@PEG, we performed cytoskeleton costaining and TEM in P1G1 cells. Confocal microscopy revealed robust nanoparticle uptake after 24 h, with PDA@PEG (visible as dark bright-field particles) localized primarily in the cytoplasm. TEM analysis further confirmed these findings, as distinct PDA@PEG nanoparticles were captured within intracellular vesicles and cytoplasmic compartments at 24 h post-incubation (Fig. 3O-P). Phalloidin-stained cells maintained an intact morphology and cell‒cell junctions throughout the incubation period. Strikingly, compared with H₂O₂-injured cells, PDA@PEG monotherapy significantly increased melanin synthesis by 1.32-fold (*p* < 0.05), whereas G-Exos alone had no such effect (Fig. 3Q). Unexpectedly, the G-Exo/PDA@PEG combination further potentiated melanogenesis by 1.41-fold (*p* < 0.01). These results show that the synergy extends beyond cytoprotection to melanin production. The data suggest that by reducing oxidative stress that inhibits melanogenesis, G-Exos enhance PDA@PEG-driven melanin production, supporting the therapeutic value of this combination.

### Characterization of G-Exos@TM and the fabrication and evaluation of HAMA/G-Exo@TM/PDA@PEG MNs

TM, an FDA-approved compound, can encapsulate hydrophobic molecules through van der Waals interactions by forming micelles [39]. In this study, TM was employed to encapsulate G-Exos to serve dual purposes: to protect the stability of G-Exos during delivery and to leverage its enzyme-responsive properties for targeted release at the lesion sites. This design was based on the established pathological feature of vitiligo, namely the overexpression of MMP-9 in the affected areas [26]. Given that the ester bonds in TM are known substrates for MMP-9 [40], we anticipated that the TM coating would be degraded specifically in the inflammatory microenvironment with elevated MMP-9 levels, thereby enabling the localized release and concentration of G-Exos activity at the target site.

**Fig. 4:**
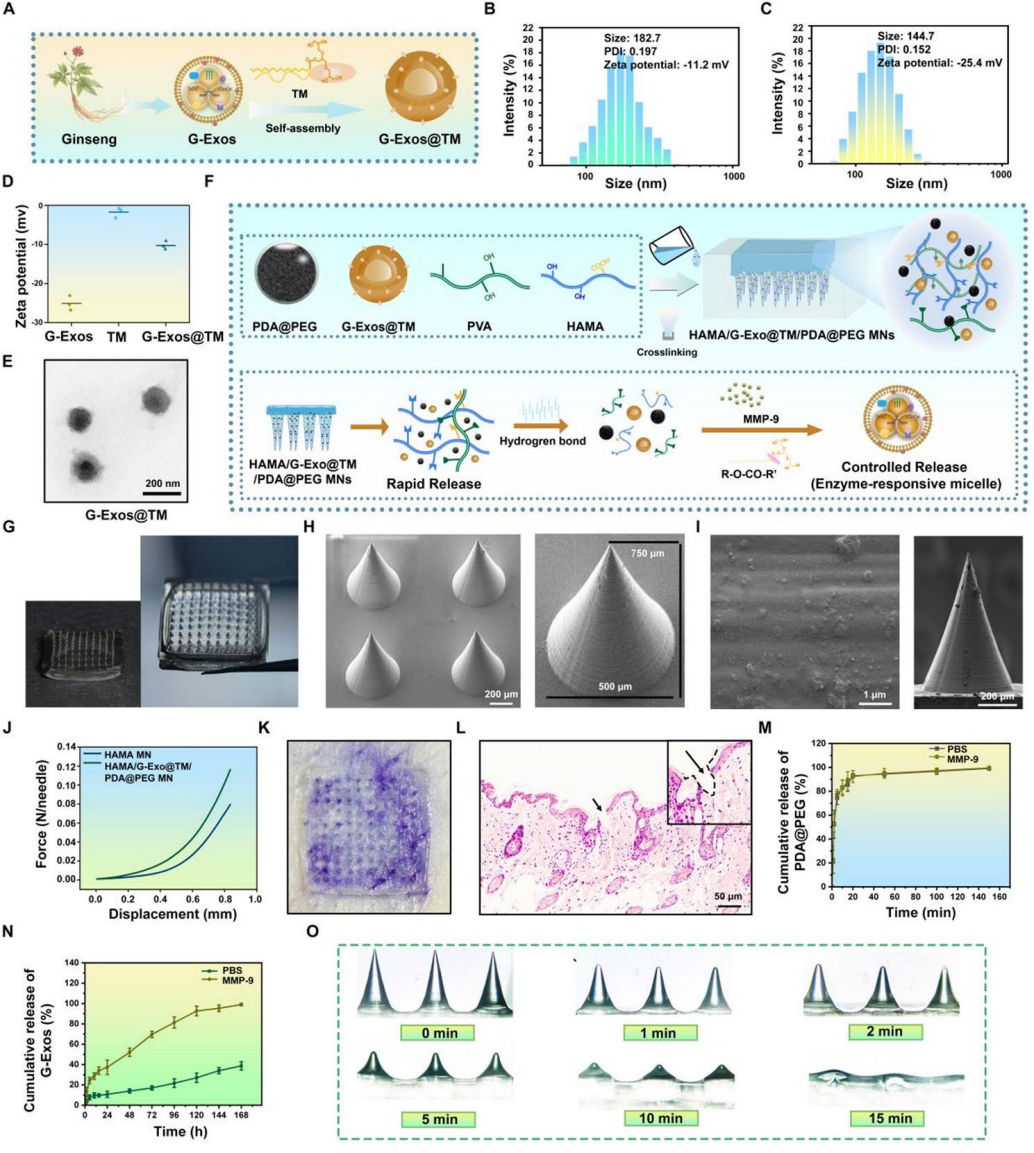
Characterization of G-Exos@TM and the fabrication and evaluation of HAMA/G-Exo@TM/PDA@PEG MNs. (**A**) Schematic illustration of the self-assembly process of G-Exos@TM. (**B**) Hydrodynamic size of G-Exos@TM. (**C**) Hydrodynamic size of G-Exos. (**D**) Zeta potentials of G-Exos, TM, and G-Exos@TM. (**E**) Representative TEM image of G-Exos@TM. (**F**) Schematic diagram of the fabrication process of HAMA/G-Exo@TM/PDA@PEG MNs and its smart stimuli-responsive release mechanism. (**G**) Frontal and lateral view photographs of the microneedle. (H) Representative SEM image of the microneedle patch from an oblique view. (I) Surface topography and Oblique view of the array. (**J**) Stress‒displacement curves from axial compression tests comparing the mechanical strength of plain HAMA MNs and HAMA/G-Exo@TM/PDA@PEG MNs. (**K**) Retention of blue-dyed microneedle tips in mouse skin. (**L**) H&E-stained skin tissue after MN penetration. (**M-N**) In vitro cumulative release of (**M**) PDA@PEG and (**N**) G-Exos from HAMA/G-Exo@TM/PDA@PEG MNs in PBS or MMP-9 solution (*n* = 3). (**O**) Dissolution processes of the microneedles

TMs self-assembled around G-Exos in aqueous solution, forming uniform micellar structures (Fig. 4A). Successful encapsulation was confirmed by several techniques. The hydrodynamic diameter of G-Exos@TM increased to 182.7 ± 19 nm from 144.7 ± 15 nm for bare G-Exos (Fig. 4B-C). Zeta potential measurements provided further evidence: while TM itself was nearly electroneutral (− 1.6 ± 0.2 mV) and unmodified G-Exos exhibited a potential of − 26.6 ± 2.2 mV, the G-Exos@TM complex showed an intermediate value of − 11.2 ± 1.2 mV (Fig. 4D). This moderate negative charge is advantageous for the electrostatic adsorption of G-Exos@TM to positively charged sites within the inflammatory microenvironment, potentially enhancing its retention and localized accumulation [41]. TEM revealed a vesicle-like structure surrounded by a distinct membrane, which was consistent with successful micellar encapsulation (Fig. 4E).

To validate this enzyme-responsive design, we evaluated the release profile of G-Exos@TM in vitro in the presence of MMP-9. TEM imaging confirmed that the released vesicles maintained intact morphology (Fig. S10). DLS analysis revealed nearly complete release of G-Exos after 7 days in the MMP-9 solution, with the particle size reverting to that of native G-Exos. In contrast, the particle size decreased only slightly in PBS without MMP-9, remaining at approximately 170 nm (Fig. S11A). Using a TINGO exosome ELISA kit, we quantified the release and found that less than 7% of the G-Exos were released over 7 days in PBS, indicating high stability without significant burst release. However, in the presence of MMP-9, nearly all the G-Exos were released by day 7 (Fig. S11B). These results collectively confirm that G-Exos@TM possesses excellent enzyme-responsive properties, enabling on-demand release of G-Exos under elevated MMP-9 conditions, such as those found in vitiligo.

Building upon the established use of HAMA MNs for transdermal drug delivery [42, 43], we integrated the enzyme-responsive G-Exos@TM into this system to achieve temporally controlled spatiotemporal release. The PDA@PEG nanoparticles and G-Exos@TM complexes were co-blended into a HAMA precursor, mixed with PVA, and photocrosslinked to form a tough double-network hydrogel. This matrix was chosen to address functional requirements not easily met with single-component systems. The covalent HAMA network contributes structural integrity, while the physical PVA network introduces flexibility. This structure facilitates the rapid diffusion of small PDA@PEG nanoparticles upon hydration, while the denser network restricts the larger G-Exos@TM complexes, enabling their sustained release until enzymatic activation occurs. This principle of using a double-network to differentially regulate diffusion based on solute size and network density is supported by work on similar HAMA-PVA systems [44].

The release mechanism is designed to be stimuli-responsive: G-Exos are encapsulated in TM micelles via hydrophobic interactions, preventing premature leakage. In MMP-9-rich vitiligo skin, the ester bonds in the TM are hydrolyzed, triggering targeted exosome release. Concurrently, the PDA@PEG nanoparticles are rapidly released for prompt therapy, while the HAMA matrix provides supplementary hydrogen bonding to synergistically prevent burst release and ensure precise, MMP-9-activated delivery (Fig. 4F). The rapid swelling and dissolution of the MNs is a characteristic feature of fast-dissolving microneedle systems [45, 46], which is engineered to facilitate the quick release of PDA@PEG nanoparticles.

The structural integrity of the microneedle patch was evaluated at multiple scales using stereomicroscopy and SEM. The stereomicroscope image revealed a densely packed, uniform, and well-aligned microneedle array (Fig. 4G). Further SEM analysis confirmed that the microneedles possessed a consistent conical morphology with sharp tips and a smooth, homogeneous surface texture (Fig. 4H-I). To ensure drug delivery to the dermis where melanocytes reside, the needles were designed with a tip height of 750 μm and a base width of 500 μm, sufficient to bypass the stratum corneum (10–40 μm) and epidermis (40–1600 μm). Mechanical testing revealed that the fracture force of the HAMA/G-Exo@TM/PDA@PEG MN patch was 0.13 N/needle, which is greater than the reported minimum skin insertion force of 0.1 N/needle [47], indicating sufficient mechanical strength for skin penetration (Fig. 4J). Experimental validation of skin penetration was performed. Optical imaging of trypan blue-stained mouse skin after a 10-minute patch application showed that most microneedle tips created visible microchannels, confirming successful penetration (Fig. 4K). H&E staining corroborated these results, clearly showing that the microneedles fully penetrated the stratum corneum (indicated by black arrows in Fig. 4L).

The release profiles from the MN system were evaluated. First, the PDA@PEG nanoparticles exhibited rapid and sustained release, reaching 98.4% within 2 h in both PBS and MMP-9 solution, with no significant difference (Fig. 4M). In contrast, the release of G-Exos was enzyme-triggered. In the presence of MMP-9, the cumulative release reached 71.3% by day 3 and 99.4% by day 7, whereas in PBS alone, it was significantly slower, with only 18.5% and 38.2% released at the same time points, respectively (Fig. 4N). We then assessed the swelling behavior of the MNs, which is crucial for drug release, in PBS (pH 7.4) at 37 °C. The MNs absorbed water and swelled, undergoing progressive structural loss over time, with the shape almost collapsing after 15 min (Fig. 4O), a phenomenon essential for facilitating steady transdermal drug release from the hydrogel matrix [48]. Internal SEM images of microneedles showed a transition from a dense, fine-porous structure at 0 min to an expanded, coarse-porous architecture after 15 min in PBS, confirming rapid matrix hydration (Fig. S12). The distinct release profiles of PDA@PEG and G-Exos correspond to their different therapeutic roles. While PDA@PEG provides early pigmentary effects, the sustained release of G-Exos supports prolonged microenvironmental regulation. The combination appears to generate a functional cascade where initial intervention creates conditions conducive to longer-term repair. Finally, a CCK-8 assay demonstrated that the MNs had no significant cytotoxic effects on P1G1 or HaCaT cells compared with the untreated control (Fig. S13). In conclusion, this study presents more than just the application of two known agents. We have introduced and validated a comprehensive therapeutic strategy that integrates G-Exos and PDA@PEG into a smart MN platform. The demonstrated synergistic effects in antioxidant, anti-inflammatory, and melanogenic activities confirm the necessity of the combination. Furthermore, the successful engineering of a system that provides spatiotemporally orchestrated drug release addresses a key limitation in current topical treatments. By concurrently targeting the multiple pathological axes of vitiligo through a single, intelligent device, this work represents a significant conceptual and technological advance with strong potential for clinical translation. Taken together, we successfully constructed an enzyme-responsive MN platform for the spatiotemporally controlled co-delivery of G-Exos and PDA@PEG. The system demonstrated MMP-9-responsive release, suitable mechanical strength for skin penetration, and good biocompatibility. We next evaluated its therapeutic efficacy in a vitiligo animal model.

### Vitiligo modeling, therapeutic intervention, and response assessment

**Fig. 5:**
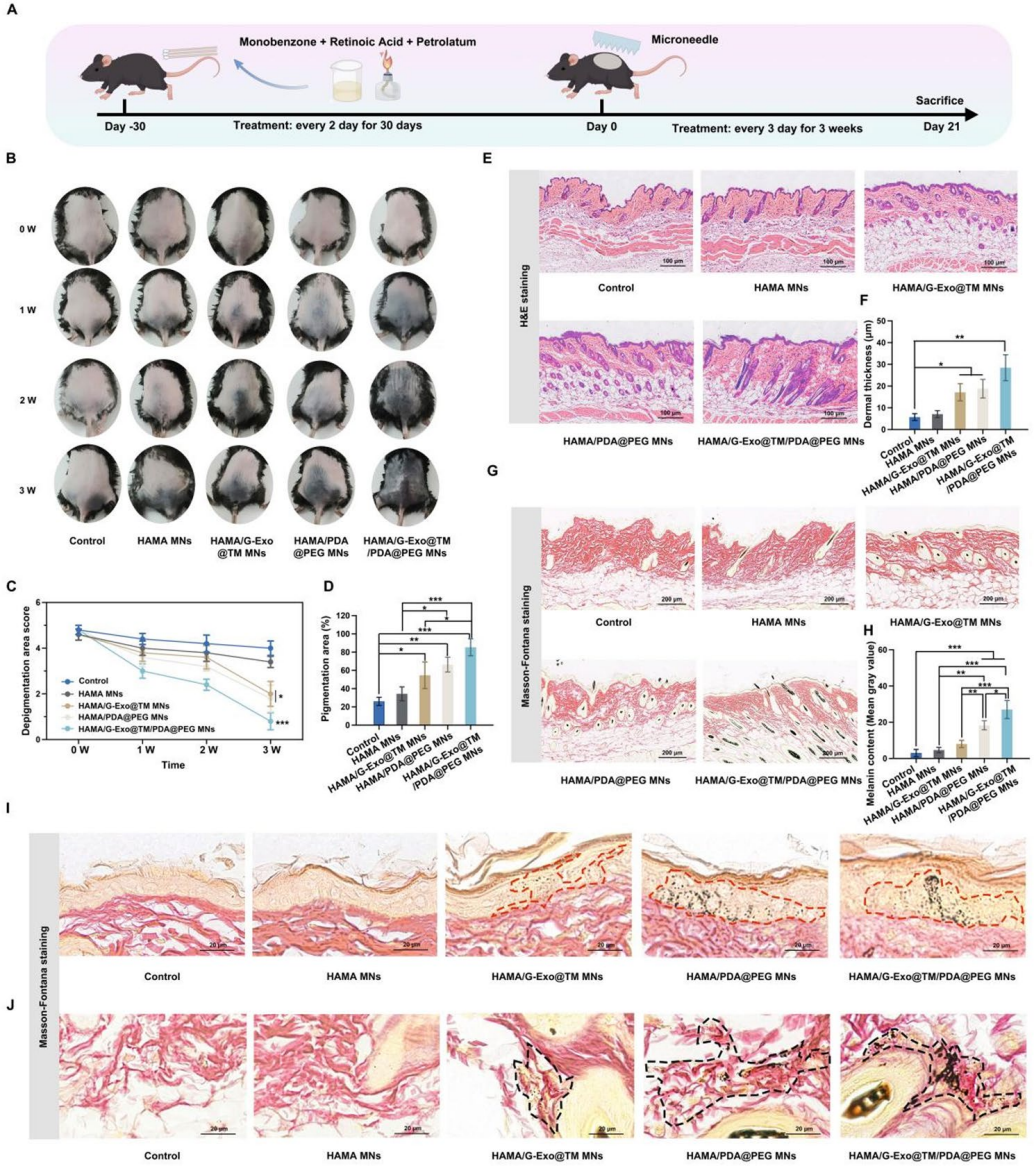
Vitiligo modeling, therapeutic intervention, and response assessment. (**A)** Schematic of the modeling and treatment protocol. (**B**) Representative photographs of depigmented skin areas after modeling and weekly treatment. (**C**) Vitiligo treatment scoring system: 0% (0 points); >5% (1 point); >5–25% (2 points); >25–50% (3 points); >50–75% (4 points); >75–100% (5 points). (**D**) Statistical analysis of the pigmentation rate after 3 weeks of treatment. (**E**) Representative H&E-stained skin tissue after 3 weeks of treatment. (**F**) Quantitative analysis of dermal thickness across groups. (**G**) Representative Masson-Fontana-stained skin showing melanin. (**H**) Quantitative analysis of melanin content in hair follicles across groups. (**I**) Representative Masson-Fontana staining images of epidermal melanin (the red areas indicate melanin granules). (**J**) Representative Masson-Fontana staining image demonstrating melanin granule transport from hair follicles to surrounding dermal cells (black areas indicate transported melanin granules). Data are presented as the means ± SD: *p < 0.05, **p < 0.01, ***p < 0.001

A stable vitiligo phenotype was successfully established in C57BL/6 mice through 30-day alternate-day topical application of 40% monobenzone/0.1% retinoic acid emulsion, which induced selective follicular melanin loss while maintaining epidermal integrity, closely mimicking human vitiligo pathology [49]. On day 30 post-induction, the depigmented dorsal zones received HAMA/G-Exo@TM/PDA@PEG MNs via patch application every other day for three weeks, with weekly photographic documentation of dorsal hair depigmentation changes and post-sacrifice histological examination (Fig. 5A). Throughout the treatment period, a single circular microneedle patch was centered on the depigmented dorsal area during each application. Treatment efficacy progressed over time. At week 1, G-Exo/PDA@PEG-treated mice exhibited significant hair repigmentation within the application zones, showing a more pronounced effect compared to other groups. By week 2, this therapeutic advantage intensified-combination therapy demonstrated markedly superior repigmentation versus individual monotherapies or empty MNs. At week 3, nearly complete repigmentation was achieved with the G-Exo/PDA@PEG composite. Remarkably, even empty MN patches alone triggered repigmentation (~ 20%) by week 3. Notably, blank microneedle patches alone induced approximately 20% repigmentation at this endpoint—a phenomenon previously documented with physical microneedle stimulation in vitiligo models [50]. The control groups maintained extensive, stable depigmentation throughout the study (Fig. 5B). These observations were corroborated by vitiligo scoring: All groups initially presented scores > 4 (> 50% dorsal depigmentation). While the control group maintained scores of ~ 4 throughout the experiment, the scores of the G-Exo/PDA@PEG-treated mice progressively improved to ~ 2 at week 2 and further decreased to 1 by week 3 (Fig. 5C). ImageJ quantification confirmed a significant increase in the repigmented surface area compared with that of the model controls at week 3 (*p* < 0.001; Fig. 5D). Notably, microneedle application alone induced modest repigmentation, which is consistent with reports that physical stimulation via microneedles activates canonical Wnt signaling to upregulate melanogenic genes (MITF, TRP1, TRP2, tyrosinase) [51]. This finding validates the intrinsic therapeutic potential of microneedling while demonstrating enhanced efficacy when it is leveraged for drug co-delivery.

H&E staining of the dorsal skin revealed that the G-Exo/PDA@PEG MN-treated groups presented significantly increased hair follicle density, with follicles predominantly in the late anagen phase and substantial melanin deposition within follicles (Fig. 5E). In contrast, compared with the control group, the G-Exo and PDA@PEG monotherapy groups presented increased follicle numbers, but these follicles were arrested primarily in the early anagen phase. The measurements revealed significant dermal thickening: the dermal thickening in both monotherapy groups exceeded that in the untreated/empty MN groups (*p* < 0.01), whereas combination therapy resulted in maximal dermal thickening (Fig. 5F). Masson-Fontana staining confirmed robust melanogenesis during treatment, with all MN-treated groups presented increased intrafollicular melanin synthesis and developed hair bulbs. Critically, combination-treated follicles exhibited elongated lower halves and maximal melanin content, whereas the untreated/empty MN groups maintained sparse follicular structures with minimal melanin granules (Fig. 5G-H). This synergistic effect may arise from dual-pathway modulation: G-Exos likely mitigate the oxidative microenvironment by scavenging reactive oxygen species, which could create more permissive conditions for melanocyte regeneration, whereas PDA@PEG provides exogenous melanin-like polymers that may facilitate pigment deposition.

Vitiligo manifests as skin depigmentation due to epidermal melanin deficiency [52], although critical interspecies differences exist: Human skin coloration arises from melanosome transfer from basal-layer melanocytes to keratinocytes, whereas C57/BL6 mice exhibit epidermal melanocyte deficiency—their melanocytes reside primarily in hair follicles, resulting in pink skin and follicular-predominant pigmentation. Therefore, while murine depigmentation typically manifests as hair whitening rather than epidermal macules with minimal observable epidermal melanin granules [53], our interventions induced substantial melanin accumulation throughout the epidermal layer (red area, Fig. 5I). Both G-Exos and PDA@PEG significantly enhanced epidermal melanin deposition, with G-Exo/PDA@PEG microneedles additionally stimulating abundant follicular melanin synthesis and transport to adjacent cells (black area, Fig. 5J). These findings demonstrate the dual capacity of PDA@PEG to supplement melanin exogenously while facilitating melanosome trafficking, confirming effective transdermal pigmentation restoration. The microneedle platform was crucial for this outcome. The creation of microchannels through the stratum corneum enables enhanced drug diffusion [48]. Furthermore, the hydrogel formulation allowed for sustained release, reducing the administration frequency [54]. This localized approach achieved superior efficacy at minimal drug concentrations, avoiding systemic toxicity from oral therapies and reducing adverse events [55, 56].

### Evaluation of antioxidant and anti-inflammatory effects in vitiligo model

**Fig. 6:**
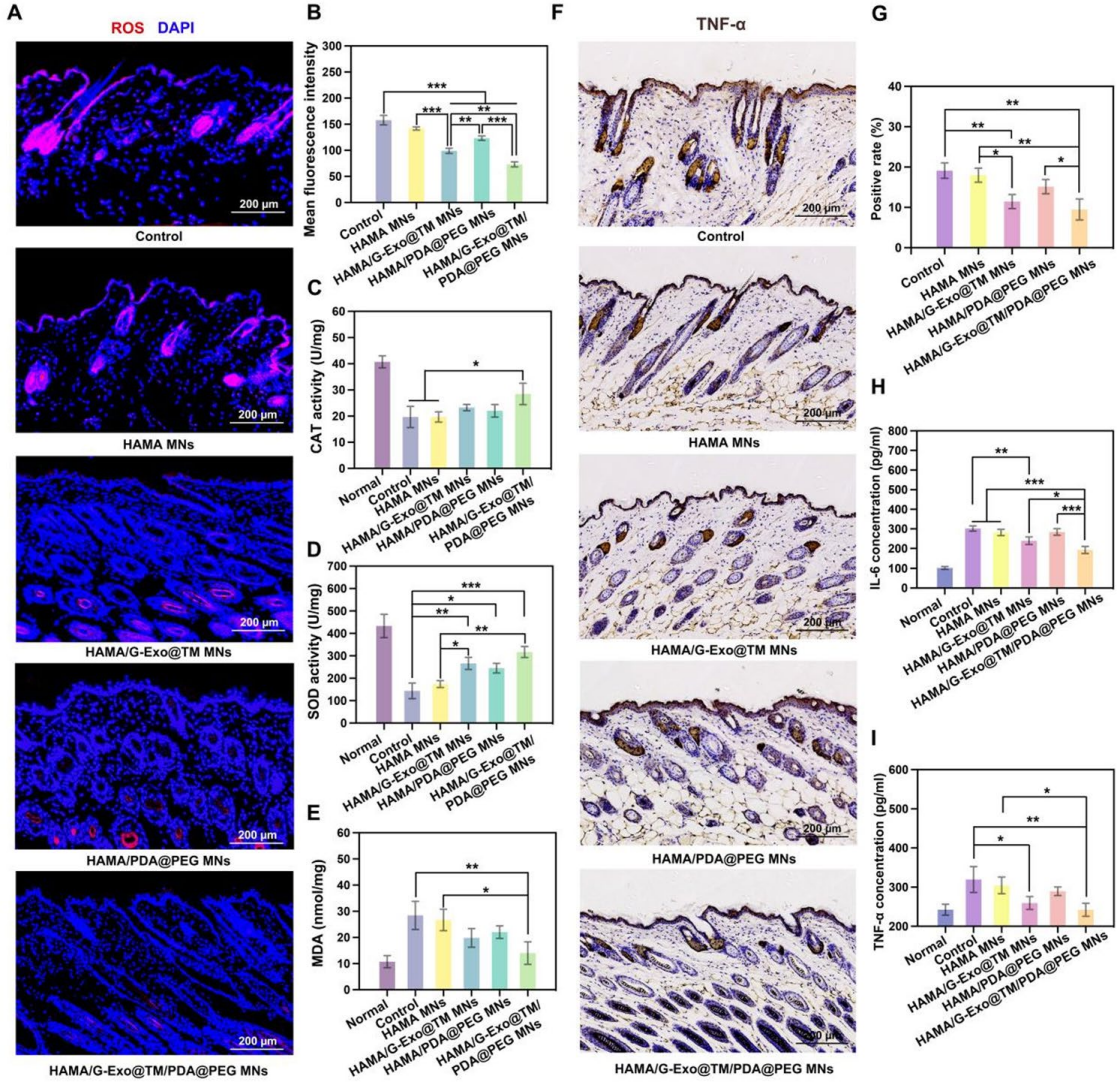
Evaluation of antioxidant and anti-inflammatory effects in vitiligo model. (**A-B**) Representative immunofluorescence images (blue: DAPI; red: ROS) (**A**) and quantification of the ROS intensity (**B**). (**C**) CAT activity, (**D**) SOD activity, and (**E**) MDA content in skin tissues after 3 weeks of treatment. (**F-G**) Representative immunohistochemical staining of TNF-α in the skin (**F**) with corresponding quantitative analysis of TNF-α expression levels (**G**). (**H-I**) IL-6 (**H**) and TNF-α (**I**) concentrations in skin tissue homogenates following 3 weeks of treatment. Data are presented as the means ± SD: *p < 0.05, **p < 0.01, ***p < 0.001

Monobenzone, a phenolic compound, induces melanocyte depigmentation primarily through oxidative stress and apoptosis. Retinoic acid potentiates this process by increasing epidermal turnover and stimulating the production of pro-inflammatory cytokines (IL-6 and TNF-α). In vivo evaluation revealed significant ROS accumulation in monobenzone/retinoic acid-treated skin, which was effectively mitigated by HAMA/G-Exo@TM/PDA@PEG MNs (Fig. 6A-B).

Oxidative marker analysis revealed that vitiligo patient skin exhibited sharply reduced SOD/catalase activity and elevated MDA levels. These pathological changes were reversed across all treatment groups, with the G-Exos/PDA@PEG combination showing the most significant restoration of the antioxidant capacity (Fig. 6C-E). This superior efficacy likely stems from the synergistic action of both components: while PDA@PEG contributes to radical scavenging, G-Exos provide robust cytoprotection and demonstrate a stronger capacity for neutralizing ROS and alleviating oxidative damage. Post-treatment cytokine assessment revealed significant TNF-α and IL-6 suppression in the G-Exos and combination groups, with PDA@PEG monotherapy showing limited effects (Fig. 6F-I). The combined G-Exo/PDA@PEG treatment showed superior efficacy to either treatment alone, which was consistent with our in vitro results. Furthermore, a significant decrease in MMP-9 expression was observed following MN treatment (Fig. S14). This reduction correlates with successful drug release and therapeutic efficacy, offering supportive indirect evidence that drug release from the TM system is likely activated by the high MMP-9 levels present in diseased skin.

### Effects on hair follicle stem cell proliferation and melanocyte function

**Fig. 7:**
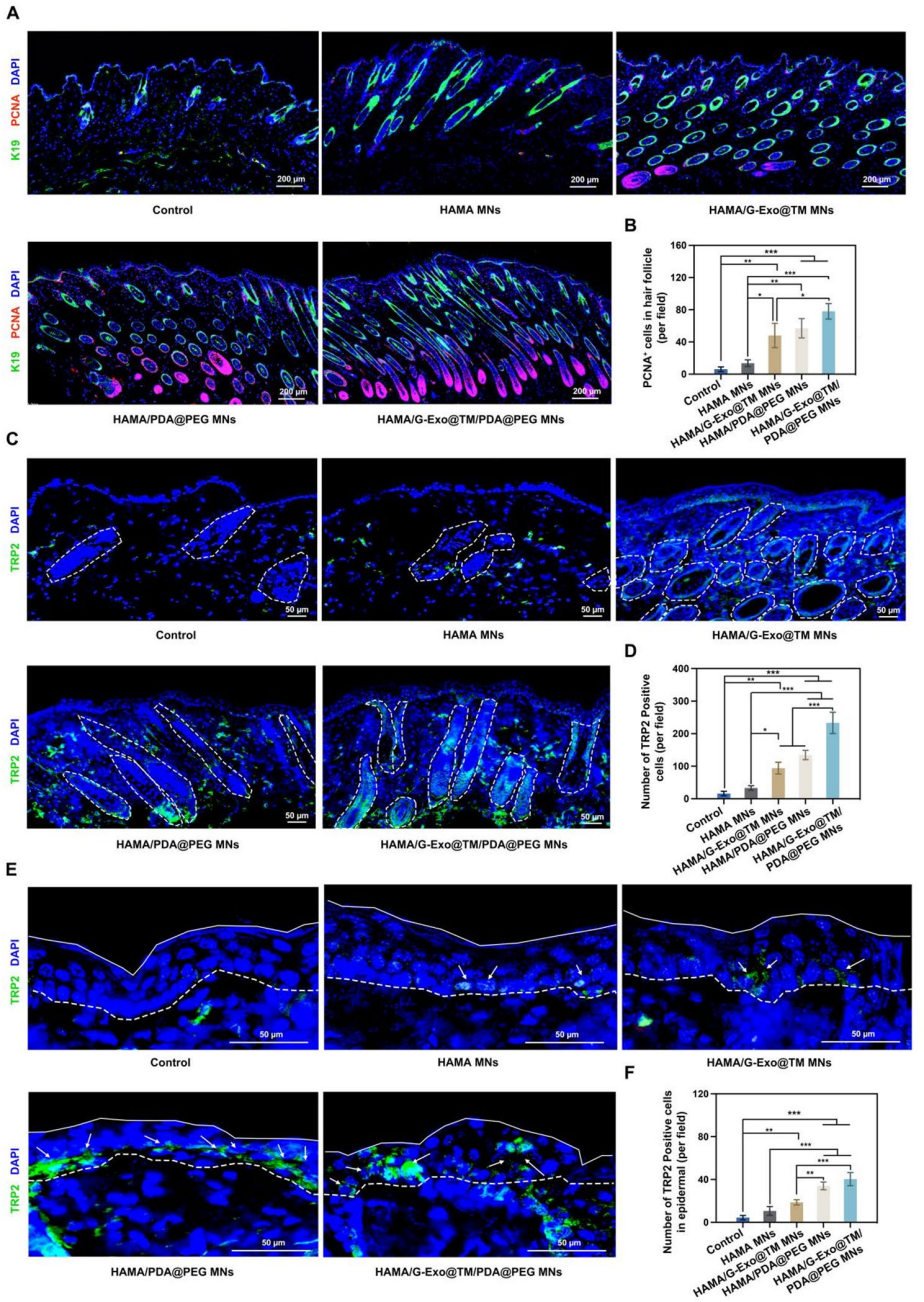
Effects on hair follicle stem cell proliferation and melanocyte function. (**A-B**) Immunofluorescence double staining for PCNA (green) and the hair follicle stem cell marker K19 (red) (**A**), which was quantified in (**B**) for proliferative cells within hair follicles. (**C**) TRP2 (melanin synthesis marker) expression in hair follicles (white dashed outlines) with quantitative analysis of positive cells per group (**D**). (**E**) TRP2 staining in the epidermis (white dashed outlines) with quantitative analysis of the positive epidermal area per group (**F**). Data are presented as the means ± SD: *p < 0.05, **p < 0.01, ***p < 0.001

Previous studies have demonstrated that hair follicle stem cells play a key role in regulating hair growth and melanin-dependent pigmentation [51]. To assess this effect, proliferating hair follicle stem cells were identified via dual immunofluorescence staining for K19 (a stem cell marker) and PCNA (a proliferation marker). While minimal K19^+^/PCNA^+^ cells were observed in the blank microneedle control group, all the therapeutic groups exhibited significant co-localization, with the most pronounced signal in the G-Exo/PDA@PEG-treated group (Fig. 7A-B; *p* < 0.001). TRP2 immunofluorescence analysis (a melanocyte-specific marker) revealed a substantially greater density of functional melanocytes in the hair follicles of G-Exo/PDA@PEG-treated mice than in those of untreated controls (Fig. 7C-D; *p* < 0.001). Additionally, epidermal melanin deposition increased by 4.8-fold (Fig. 7E-F), which may be driven by two complementary mechanisms: PDA@PEG nanoparticles likely facilitate exogenous melanin supplementation through biomimetic deposition while simultaneously enhancing endogenous melanogenesis by stimulating follicular melanocyte proliferation. Moreover, G-Exos contributed to a supportive microenvironment by suppressing oxidative stress and inflammation, which appears to be crucial for maintaining PDA@PEG-induced melanogenesis. Together, these findings highlight the ability of the combination strategy to achieve both rapid pigmentation and sustained melanocyte regeneration.

### RNA-seq analysis of the mechanism of action of the MN system

**Fig. 8:**
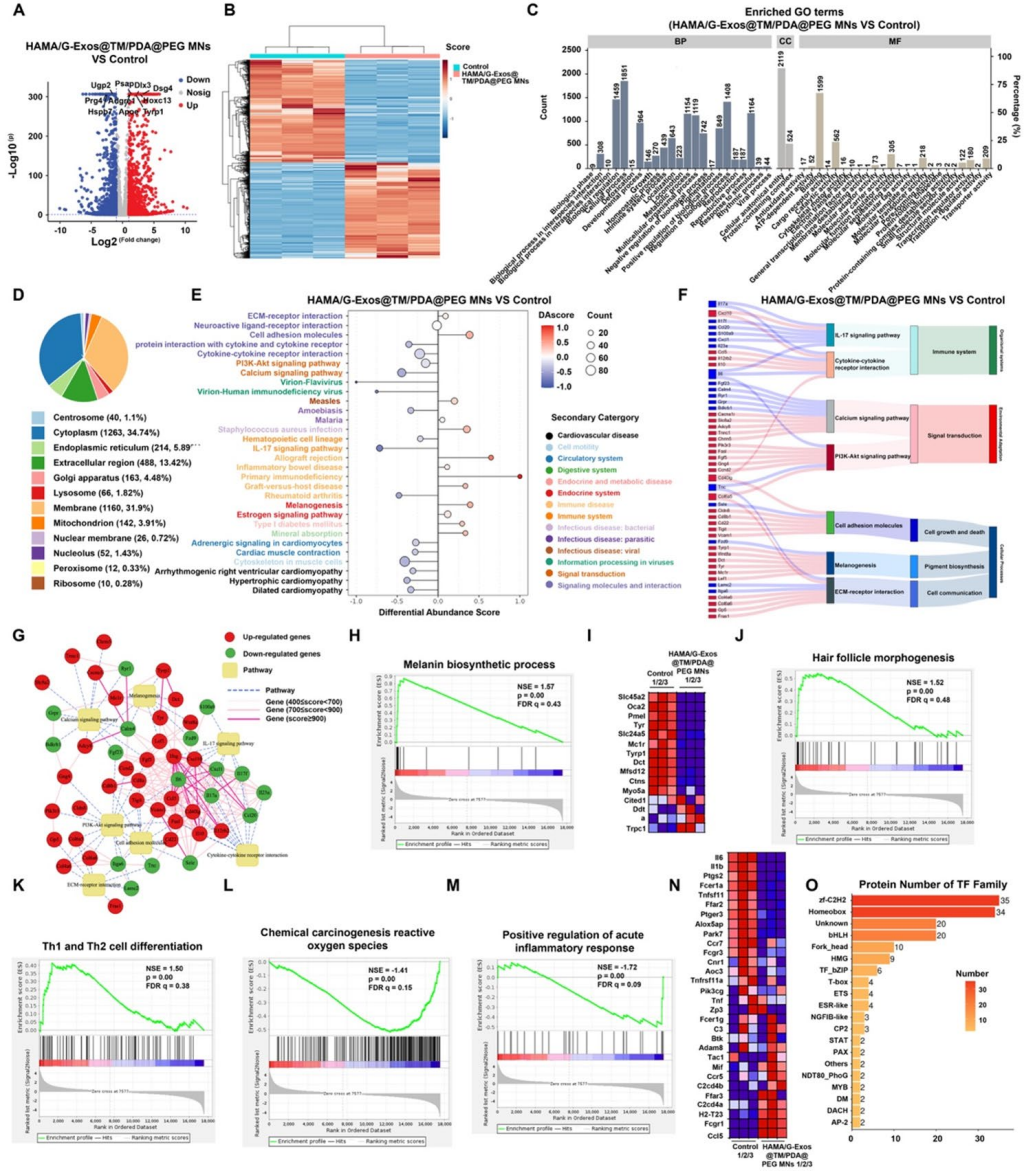
RNA-seq analysis of the mechanism of action of the MN system. (**A**) Volcano plot of DEGs between the G-Exo/PDA@PEG MMP-treated group and the control group. (**B**) PCA plot demonstrating clustering of biological replicates (*n* = 3 per group). (**C**) GO level 2 functional enrichment of DEGs. (**D**) GO cellular component (CC) subcellular localization analysis of DEGs. (**E**) DA score-based trend analysis of the top 30 enriched KEGG pathways (secondary hierarchy) for DEGs. (**F**) Sankey diagram showing selected KEGG pathways of interest (from the top 30), associated DEGs, and their top class/second class classifications. (**G**) PPI network of key genes from the pathways in (**F**). (**H–I**) Enrichment plots from GSEAs of gene sets for the “Melanin biosynthetic process” gene set. (**J–L**) GSEA plots for the “Hair follicle morphogenesis,” “Th1/Th2 cell differentiation,” and “Chemical carcinogenesis-reactive oxygen species” gene sets. (**M–N**) Enrichment plots from GSEAs of gene sets for the “Positive regulation of acute inflammatory response”. (**O**) TF families predicted from DEG promoter motifs

To elucidate the molecular mechanisms underlying the therapeutic effects of the multifunctional hydrogel system incorporating G-Exos/PDA@PEG, we performed RNA sequencing on skin tissue from vitiligo model C57BL/6 mice. This analysis identified 2,977 differentially expressed genes (DEGs) in the G-Exo/PDA@PEG treatment group versus the control group, with 1,377 upregulated and 1,600 downregulated genes. Notably, melanogenesis is activated through the upregulation of Dlx3 (a direct Tyrp1 activator) and hair follicle repair genes (Hoxc13, Dsg4); anti-inflammatory effects are mediated by the downregulation of microenvironment modulators (Prg4, Hspb7); and oxidative stress resolution via ApoE upregulation (enhancing antioxidant defense) coupled with Prg4 suppression (reducing ROS-mediated NLRP3 activation), collectively demonstrating the system’s multi-target approach to skin repigmentation (Figs. 8A-B, S15-S16). Genomic circos plots revealed the spatial organization of these DEGs, providing dimensional insights into treatment-induced transcriptional reprogramming (Fig. S17). GO analysis revealed significant enrichment of DEGs involved in immune system processes and pigmentation pathways (Fig. 8C). Subcellular localization analysis revealed cytoplasmic and membrane clustering of the DEGs, including upregulated Psap and ApoE in the cytosol. This finding is consistent with cellular uptake mechanisms that G-Exos undergo endocytosis and engage membrane receptors, whereas PDA@PEG disperses diffusely within the cytoplasm after uptake (Figs. 3P and 8D). KEGG pathway trend analysis via differential abundance (DA) scores (top 30 pathways) further revealed that the upregulated genes were enriched in ECM‒receptor interactions, cell adhesion molecules, and melanogenesis, whereas the downregulated genes were enriched in cytokine‒cytokine receptor interactions, PI3K/AKT signaling, calcium signaling, and IL-17 pathways (Fig. 8E). Sankey diagram visualization highlighted key gene‒pathway relationships, with the widest downregulation flows involving inflammatory mediators (TNC, IL-6, and IL-17a) and the strongest upregulation flows featuring immunomodulatory/repair factors (IFNG, CXCL10, CD40LG, and COL6A5), suggesting potential interactions between inflammatory and repair axes (Fig. 8F). Protein interaction network analysis of these pathways revealed critical hubs and modular functions (Fig. 8G). Collectively, these findings suggest the establishment of a synergistic “repair-anti-inflammatory” network: wherein ECM reconstruction and enhanced cell adhesion may create a microenvironment that is supportive of melanocyte survival, whereas the initial suppression of inflammation (via IL-17/cytokine downregulation) precedes regenerative activation (melanogenesis upregulation). Gene set enrichment analysis (GSEA) confirmed significant upregulation of melanin biosynthesis, hair follicle morphogenesis, and Th1/Th2 differentiation pathways, along with downregulation of ROS-mediated chemical carcinogenesis and acute inflammatory response pathways (Fig. 8H-N, S18-S20). Key transcription factor families (homeobox, zf-C2H2, bHLH, and Fork_head/HMG) collectively orchestrate melanogenesis activation, ECM remodeling, and anti-inflammatory responses, establishing a pro-regenerative microenvironment (Fig. 8O). Key findings from the transcriptome analysis were validated at the protein level via immunofluorescence staining and Western blot analysis for IL-17 A, PI3K, and ApoE. The results (Fig. S21-S22) were consistent with the RNA-seq data, showing a significant reduction in IL-17 A and an increase in PI3K and ApoE in the treatment group.

Integrating the RNA-seq data with supporting evidence from protein-level analyses (Fig. S21-S23), we propose a multi-faceted mechanism of action. The composite MN patch is suggested to promote repigmentation through coordinated anti-inflammatory effects (notably G-Exo-mediated IL-17 A suppression), activation of pro-melanogenic pathways (e.g., PI3K/AKT), and enhanced antioxidant defenses (e.g., ApoE upregulation). It is important to note that the precise molecular interplay, particularly the causal relationships within these pathways, requires further validation in future studies.

## Conclusion

This study developed a temporally controlled hydrogel microneedle system for the synergistic delivery of G-Exos and PDA@PEG in vitiligo treatment. The system enables rapid release of PDA@PEG upon matrix dissolution, followed by MMP-9-responsive degradation of micelles for on-demand, lesion-specific release of G-Exos. Functionally, G-Exos help restore redox homeostasis via ApoE upregulation and attenuate inflammation through IL-17 A pathway suppression, while PDA@PEG promotes repigmentation via exogenous pigment deposition and activation of the PI3K/AKT pathway to stimulate endogenous melanogenesis. In a vitiligo mouse model, this strategy achieved marked skin repigmentation within three weeks, demonstrating coordinated modulation of the oxidative stress–inflammation–melanocyte dysfunction axis via a minimally invasive approach. This work presents an integrated and intelligent therapeutic platform, providing a foundation for further translational development in vitiligo treatment.

## Supplementary Information

Supplementary Material 1.

## Data Availability

All data obtained throughout this study are presented in the manuscript or supporting information. All relevant data are available from the corresponding author upon reasonable request.
