# Supplementary material for: Synergistic delivery of ginseng exosomes and biomimetic melanosomes via temporally controlled hydrogel microneedles restrain the pathologic triad of vitiligo
Source: J Nanobiotechnology. 2026 Mar 2;24:325. doi: 10.1186/s12951-026-04168-w (PMC13059390; doi:10.1186/s12951-026-04168-w)
Supplement: Supplementary file 1 — Supplementary Material 1. [file 12951_2026_4168_MOESM1_ESM.docx]

**Materials and methods**

**Materials and animals**

Sucrose and sodium hydroxide (NaOH) were obtained from Aladdin (Shanghai, China). Dopamine hydrochloride, mPEG5000-NH₂, EDTA, paraffin, histological staining reagents, and the nuclear stain 4’,6-diamidino-2-phenylindole (DAPI) were acquired from Sigma‒Aldrich (USA). Hyaluronic acid methacrylate (HAMA), the photoinitiator lithium phenyl-2,4,6-trimethylbenzoylphosphinate (LAP), polyvinyl alcohol (PVA), and triglycerol monostearate (TM) were obtained from Engineer For Life (Beijing, China) and Macklin (Shanghai, China). The human epidermal melanocyte cell line PIG1 was generously provided by Dr. Chunying Li (Xijing Hospital, Xi’an, China), and the HaCaT keratinocyte cell line was procured from the Cell Resource Center of China Science Academy (Shanghai, China). Cell culture reagents—including fetal bovine serum (FBS), penicillin‒streptomycin (P/S), human melanocyte growth supplement (HMGS-2), and Medium 254—were obtained from Gibco (USA). Immunoassay kits for IL-6 and TNF-α quantification were supplied by Bio-Swamp (Wuhan, China), with primary antibodies against K19, PCNA, ROS, TRP2, IL-17A, PIK3R3, and ApoE sourced from Sigma‒Aldrich (USA). Phenylmethylsulfonyl fluoride (PMSF) was purchased from Beyotime Biotechnology (Shanghai, China). Phosphate-buffered saline (PBS) was acquired from Biosharp (Hefei, China). Female C57BL/6 mice (4–6 weeks, 16–20 g) were supplied by Chongqing Medical University Laboratory Animal Center. All solvents met analytical- or chromatographic-grade standards.

**Isolation, purification, and characterization of G-Exos**

G-Exos were isolated from ginseng roots (provided by the ginseng base of Changbaishan Mountain, Jilin Province, China). Fresh ginseng roots were washed with water to remove impurities and homogenized in 30 mL of PBS via a high-speed blender (10 min total, with 1-min intervals). The slurry was filtered through a sieve to remove debris. The filtrate was centrifuged at 2,000 × g for 20 min, and the supernatant was further centrifuged at 10,000 × g for 60 min. After filtering through a 0.45-μm pore membrane, the exosomes in the supernatant were subsequently pelleted by ultracentrifugation (Beckman XPN-100, USA) at 120,000 × g for 2 h, and the pellet was suspended in PBS. The cushion layer was purified via discontinuous sucrose density gradients (8%, 30%, 45%, and 60%) at 120,000 × g for 90 min. G-Exos were collected from the 30%–45% sucrose interface. All centrifugation steps were performed at 4°C. The exosome concentration was quantified as the protein content via a BCA assay kit (Beyotime, China). The morphology of the G-Exos was observed via transmission electron microscopy (TEM) (Talos L120C, Thermo Fisher Scientific, USA). The particle sizes and surface charges (represented by the surface zeta potential) were measured via laser diffraction spectrometry (Malvern Zeta sizer 3000HS, Malvern) (Brookhaven Instruments Corp, Holtsville, NY, USA). Transmission electron microscopy and a Zetasizer Nano ZS were used to visualize the G-Exos.

**Component analysis of G-Exos**

A combined multiomics approach was used to detect components in G-Exos, including lipids and proteins. The obtained G-Exos were sent to Shanghai Bioprofile Biotechnology Co. Ltd. for component analysis.

**1) Lipid analysis**

Lipid extraction was performed by adding butanol-methanol (1:1, 10 mM ammonium formate) and an internal standard to the thawed sample, followed by vortexing, sonication, and centrifugation. LC‒MS/MS analysis was conducted via a Nexera X2 LC‒30AD system (Shimadzu) with a C18 column (specific dimensions if available) and mobile phases of (A) 50% acetonitrile/10 mM ammonium acetate (pH 8.0) and (B) 100% acetonitrile, with gradient elution (0‒0.1 min: 90% B; 0.1‒5 min: 90‒65% B; 5‒5.3 min: 65‒0% B; 5.3‒7.2 min: 0% B; 7.2‒7.4 min: 0‒90% B; 7.4‒10 min: 90% B) at 300 μL/min and 35°C. Mass spectrometry was performed on a Triple Quad 6500+ (SCIEX) using ESI (± modes) with an ion spray voltage of ± 5500/4500 V, a source temperature of 400°C, a gas pressure of 1/2 55/60 psi, a curtain gas pressure of 35 psi, and a collision gas pressure of 8 psi, with the DP/CE optimized for individual lipids. The data were processed via multiquant with the LIPID MAPS nomenclature.

**2) Proteomic analysis**

G-Exo-containing proteins were extracted from beads via SDT buffer (4% SDS, 100 mM Tris-HCl), reduced (TCEP/CAA, 100°C, 5 min), alkylated (8 M urea, 150 mM Tris-HCl, pH 8.0), and digested with trypsin (20 ng/μL, 37°C, 16–18 h). The peptides were desalted (C18 StageTips) and analyzed via LC‒MS/MS (Q Exactive HF; Easy nLC; 60-min gradient: 5‒100% acetonitrile/0.1% formic acid). The data were processed via MaxQuant (v1.5.8.3) against UniProtKB Human (2017) with a 1% FDR.

**3) miRNA analysis**

Total RNA was extracted via TRIzol (Invitrogen) and quality-checked with a NanoDrop. For 3 μg of RNA, sequencing libraries were prepared through mRNA enrichment (poly-T beads), fragmentation (Illumina buffer with divalent cations), cDNA synthesis (SuperScript II/DNA Polymerase I), end repair/adapter ligation, size selection (400--500 bp, AMPure XP), and PCR enrichment (15 cycles). Library quality was verified via a Bioanalyzer 2100 (Agilent) before 150-bp paired-end sequencing on a NovaSeq Xplus instrument (Illumina).

**Synthesis and characterization of the PDA@PEG nanoparticles**

PDA was synthesized by vigorously stirring 360 mg of dopamine hydrochloride in 180 mL of deionized water at 50°C, followed by the addition of 1520 μL of NaOH (1 M) and reaction for 5 h. The resulting suspension was purified via repeated centrifugation (12,000 rpm, 10 min), rinsed with water, and then lyophilized. For PEGylation, 10 mg of PDA was dissolved in 5 mL of water, mixed with 100 mg of mPEG5000-NH₂ in 500 μL of water under ice-bath sonication (40 min, pulsed mode: 2 s on/3 s off), and stirred at RT for 12 h. The product was dialyzed (MWCO: 8–14 kDa, 48 h) with frequent water replacement. The nanoparticles were characterized via dynamic light scattering (DLS, Zetasizer), transmission electron microscopy (TEM, carbon-coated copper grid), scanning electron microscopy (SEM), Fourier transform infrared spectroscopy (FT-IR, Nicolet iS10, KBr pellets, 500–4000 cm^-1^), and X-ray photoelectron spectroscopy (XPS). Colloidal stability was evaluated by monitoring particle size over 8 weeks at 25°C.

**Preparation and characterization of G-Exos@TM**

A solution of TM (10 mg/mL) was prepared in ultrapure water by gentle heating in a water bath at 50–60°C followed by sonication to ensure complete dissolution. The G-Exos solution was subsequently added dropwise to the TM solution at a mass ratio of 1:5 (G-Exos:TM) under constant magnetic stirring. The mixture was allowed to react for 30 minutes at room temperature to facilitate initial self-assembly. To promote the formation of stable TM micelles encapsulating the exosomes while minimizing structural damage, the mixture was then subjected to probe sonication in an ice bath for 5 minutes. Following sonication, the resulting G-Exos@TM complex was dialyzed against PBS via a dialysis membrane (MWCO = 3,500 Da) to remove any organic solvent and unencapsulated free TM. The PBS buffer was replaced at least three times at 4-hour intervals. The morphological characteristics and size distributions of the G-Exos, TM micelles, and final G-Exos@TM complex were analyzed via TEM and DLS.

**Fabrication of HAMA/G-Exo@TM/PDA@PEG MNs**

The tip precursor solution was prepared by mixing HAMA (5 mg/mL), PVA (1 mg/mL), LAP (10 mg/mL), PDA@PEG (0.2 mg), and the as-prepared G-Exos@TM (0.2 mg) in deionized water. The mixture was poured into a plasma-cleaned PDMS mold and degassed under vacuum. The mold was then dried at 37°C for 2 h to presolidify the MN tips in a dust-free environment. Subsequently, photocrosslinking was performed under 405 nm UV light (10–20 mW cm⁻²) for 60–120 s to form methacryloyl groups. For the backing layer, a 20% (w/v) PVA solution was cast onto the base of the needle array, scraped flat, and dried in an oven at 37–45°C for 48 h. The fully dried MN patch was carefully demolded from the PDMS mold, sealed, and stored at 4°C in the dark until use.

**Evaluation of Mechanical Strength and Skin Penetration**

The mechanical strength of the MN patch was evaluated via a displacement‒force testing system (Hengyi, China) equipped with a 50 kg load cell. The microneedle tip was positioned vertically on a rigid stainless-steel platform. The sensor descended at a speed of 0.1 mm/s, starting from an initial distance of 1 cm above the tip. Once contact was established between the sensor and the microneedle tip, the displacement and force data were recorded. The speed was then reduced to 0.01 mm/s, and loading continued until the sensor had moved 800 μm.

To assess the skin penetration capability ex vivo, full-thickness mouse skin was collected. The MN patch was applied to the skin via thumb pressure for 10 mins. After removal, the skin was stained with trypan blue to allow for clear visualization of the penetration sites under a digital microscope. Following this, the skin was fixed with 4% paraformaldehyde, embedded in paraffin, sectioned, and subjected to H&E staining. The resulting sections were observed under a microscope to visualize the micropores created by the MNs.

**Morphological characterization of microneedles**

The MNs were thoroughly dried in a drying oven prior to characterization. The surface morphology and overall structure of the MNs were then examined and imaged via SEM.

**Dissolution of Microneedles**

The prepared MNs were immersed in phosphate-buffered saline (PBS, pH 7.4) at 37 °C to evaluate their morphological changes. After immersion for 1, 2, 5, 10, or 15 min, the surface morphology of the MNs was examined via a digital optical microscope.

***In vitro* drug release profile**

**1) Release from G-Exos@TM Complexes**

The enzyme-responsive release profile of G-Exos from the G-Exos@TM complexes was investigated. Freshly prepared G-Exos@TM were diluted in either PBS (pH 7.4) or PBS containing 1 ng/mL MMP-9. The mixtures were incubated at 37°C under constant agitation at 600 rpm. To directly visualize the micelle disassembly and exosome release, the morphological changes of the complexes after incubation with MMP-9 were examined using TEM. Concurrently, the hydrodynamic diameter of the particles in each group was monitored daily via DLS. For quantitative analysis, samples from both groups were centrifuged and resuspended at designated time points (days 1, 3, and 7). The amount of released exosomes was determined via a TINGO Exosome ELISA Kit, which measures the exposure of the exosome surface marker CD63, according to the manufacturer's instructions.

**2) Release from MN Patches**

The release profiles of PDA@PEG and G-Exos@TM from the MN patches were evaluated separately. MNs were placed in release media (PBS, pH 7.4, with or without 1 ng/mL MMP-9) at 37°C under gentle agitation. The supernatant was collected at predetermined time intervals. The cumulative release of PDA@PEG was quantified by measuring its characteristic absorbance at 280 nm via a microplate reader. The concentration of released G-Exos in the supernatant was determined via the TINGO Exosome ELISA Kit. After each collection, an equal volume of fresh prewarmed release medium (PBS or MMP-9 solution) was added to maintain sink conditions.

**Cell Culture, Treatment, and Cytotoxicity Assessment**

HaCaT cells were cultured in DMEM (Gibco) supplemented with 10% FBS (BI) and 1% penicillin/streptomycin (100 U/mL), while PIG1 cells were maintained in Medium 254 (Gibco) containing 5% FBS, 1% penicillin/streptomycin, and 10% HMGS-2 (Gibco). All the cells were incubated at 37°C under 5% CO₂.

To establish an oxidative stress model, both cell types were treated with H₂O₂ (0–2.4 mM) for 4 hours. Cell viability was assessed via the CCK-8 assay by adding 10 μL of reagent per well, incubating for 1 hour, and measuring the absorbance at 450 nm.

For the evaluation of nanomaterial cytotoxicity, oxidative stress-injured PIG1 or HaCaT cells (1×10⁴ cells/well) were exposed to G-Exos, PDA@PEG, or their combination for 24 hours. Cell viability was again evaluated via the CCK-8 assay. Live/dead staining was performed via calcein AM and propidium iodide (PI; KeyGEN) according to the manufacturer's instructions, with imaging conducted via an inverted microscope.

To evaluate the biocompatibility of the hydrogel composite, extracts of the HAMA hydrogel were prepared by immersing a predetermined amount of hydrogel in culture medium at a mass ratio of 1:10 (hydrogel to medium) followed by incubation at 37°C for 24 hours. After the cells were seeded in 48-well plates and allowed to adhere for 24 hours, the culture medium was replaced with hydrogel extract. After another 24 hours of incubation, cell viability was measured via the CCK-8 assay.

***In Vitro* Antioxidant and Anti-inflammatory Assessment**

For intracellular ROS detection and H₂O₂ catalytic evaluation in PIG1 melanocytes, cells (1×10⁵/well) were treated with H₂O₂ (0.8 μmol/mL) alone or combined with PDA@PEG (50 μg/mL), G-Exos (10 μg/mL), or both for 4 h, followed by DCFH-DA staining and flow cytometry/fluorescence microscopy analysis; parallel H₂O₂ catalytic capacity was measured in H₂O₂-pretreated cells incubated for 24 h with the same preparations via the Amplex™ Red assay. For antioxidant and anti-inflammatory assessment in HaCaT keratinocytes, cells pretreated with H₂O₂ (0.8 μmol/mL, 4 h) were incubated for 24 h with identical nanomaterials for quantification of malondialdehyde (MDA), superoxide dismutase (SOD), and catalase (CAT) activities, while LPS-stimulated cells (0.1 ng/mL, 24 h) were similarly treated with TNF-α and IL-6 secretion, as measured by ELISA.

**Melanin quantification**

The melanin content was quantified via a modified colorimetric method based on NaOH solubilization. Briefly, PIG1 cells were seeded in 6-well plates. After overnight incubation, the cells were treated with the indicated preparations (G-Exos, PDA@PEG, or G-Exos+PDA@PEG) for 24 h. The cells were then washed and lysed, and melanin was solubilized by adding 100 μL of 1 M NaOH. The absorbance of the solubilized melanin was measured at 490 nm via a microplate reader.

**Cellular Uptake and Ultrastructural Analysis of PDA@PEG**

PIG1 cells were seeded on 15-mm glass-bottom dishes and treated with 50 μg/mL PDA@PEG for 24 h. For confocal microscopy analysis, the cells were stained with rhodamine-phalloidin (KeyGEN) to visualize the cytoskeleton while simultaneously tracking PDA@PEG internalization via transmitted light detection mode. For electron microscopy analysis, the cells were fixed in 2.5% glutaraldehyde (4°C, overnight), postfixed with 1% OsO₄, and dehydrated through an ethanol‒acetone gradient. Following resin infiltration (acetone‒epoxy mixtures) and thermal polymerization (35‒80°C), ultrathin sections (70‒90 nm) were prepared via a Leica EM UC7 ultramicrotome and then stained with uranyl acetate and lead citrate for TEM observation of PDA@PEG–melanosome interactions at subcellular resolution.

**Animal Model and Treatment Protocol**

All animal procedures complied with the NIH Guide for the Care and Use of Laboratory Animals and were approved by the Animal Care and Use Committee of Chongqing Medical University (Approval No: IACUC-CQMU-2025-03045). A vitiligo mouse model was established via a validated depigmentation protocol. Female C57BL/6 mice (4–6 weeks old, 16–20 g) were anesthetized via intraperitoneal injection of 5% chloral hydrate (350 mg/kg). A topical emulsion containing 33.3% (w/w) Vaseline (4 g), 50% (w/w) 4-benzyloxyphenol (6 g), and 16.7% (w/w) tretinoin (2 g) was applied uniformly to the depilated area every other day for 30 days [1]. The mice were housed under standard conditions with ad libitum access to food and water throughout the study.

Following successful establishment of the vitiligo mouse model, the mice were randomly divided into five groups (n=5 per group): Control, HAMA MNs, HAMA/G-Exo@TM MNs, HAMA/PDA@PEG MNs, and HAMA/G-Exo@TM/PDA@PEG MNs. Dry MNs were inserted into the dorsal vitiliginous area (two patches per mouse). Treatments were administered every 3 days for 3 weeks. Depigmentation severity was scored on the basis of the percentage of depigmented area in the exposed dorsal skin: 0% = 0 points; >5% = 1 point; >5–25% = 2 points; >25–50% = 3 points; >50–75% = 4 points; and >75–100% = 5 points [2].

**Histological analysis**

Skin tissues were harvested and fixed in 4% buffered paraformaldehyde. After fixation, the tissues were dehydrated through a graded ethanol series (75%, 85%, 95%, and 100%), cleared in xylene, and embedded in paraffin. Sections (8 μm thickness) were prepared for staining. Histochemical analyses included H&E for general histomorphology and Masson–Fontana staining to quantify melanin deposition. Immunofluorescence staining was performed to evaluate the ROS levels, assess the co-localization of K19 and PCNA for hair follicle cell proliferation analysis, and determine the localization of TRP2 to identify melanocytes. Additionally, immunohistochemical staining was conducted to detect the protein expression levels of TNF-α, IL-17A, PIK3R3, and ApoE in skin tissues.

**Antioxidant stress and anti-inflammatory activity analysis *in vivo***

After treatment, the skin tissues were harvested for biochemical analyses. The MDA concentration and the activities of SOD and CAT were measured via commercial assay kits according to the manufacturer’s protocols. For cytokine quantification, additional tissue samples were homogenized in RIPA lysis buffer on ice, followed by centrifugation at 12,000 × g for 15 minutes at 4°C. The resulting supernatants were collected, and TNF-α and IL-6 levels were determined via ELISA kits following the manufacturer's instructions.

**Transcriptome Analysis of Skin Tissues**

Skin tissues from the control and HAMA/G-Exo@TM/PDA@PEG MN-treated groups were harvested post-treatment for transcriptomic profiling. Total RNA was extracted with TRIzol® Reagent (Invitrogen), and its concentration, purity, and integrity (RIN > 8.0, Agilent Bioanalyzer 2100) were verified. Using 1 μg of qualified RNA per sample, small RNA libraries were prepared with the NEBNext® Multiplex Small RNA Library Prep Kit (NEB #E7300) through adapter ligation (3’/5’), reverse transcription (SuperScript™ II), and PCR amplification. The libraries met the following QC criteria: insert size 140–150 bp (Agilent 2100 Bioanalyzer) and sufficient concentration (Qubit® dsDNA HS Assay). Pooled libraries were sequenced on an Illumina NovaSeq 6000 system (150 bp PE, 20 M reads/sample) by Shanghai Bioprofile Biotechnology Co. Ltd.

**Western Blotting**

Protein samples were resolved by SDS-PAGE, transferred to PVDF membranes, and blocked with 5% non-fat milk. Membranes were probed with primary antibodies (IL-17A, PI3K, ApoE; all from Cell Signaling Technology) at 4°C overnight, followed by incubation with HRP-conjugated secondary antibodies at 37°C for 1 h. Signal detection was performed using an ECL system. GAPDH served as the loading control.

**Statistical analysis**

All the quantitative data are presented as the means ± standard deviation (SD). Comparisons between two groups were analyzed via Student’s t test. For multigroup comparisons, one-way analysis of variance (ANOVA) followed by Tukey's post hoc test was performed via GraphPad Prism software (version 10.0). Statistical significance was defined as **p* < 0.05, ***p* < 0.01, and ****p* < 0.001.


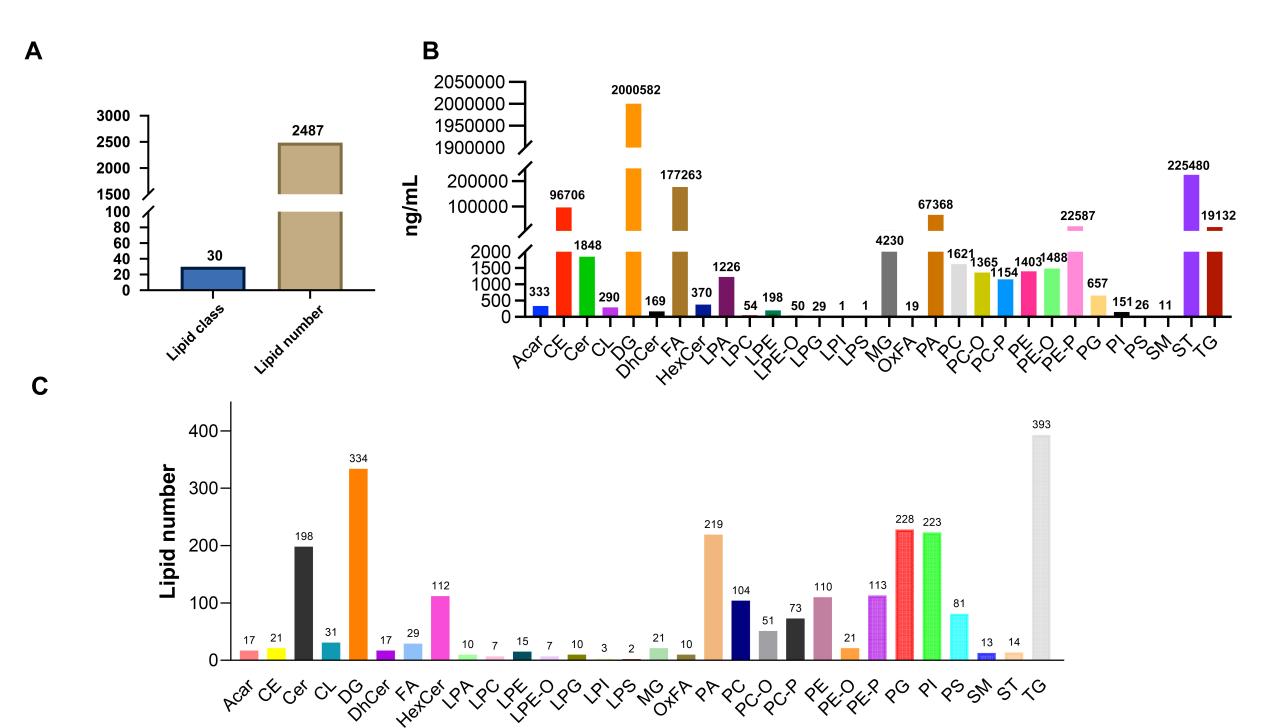


**Figure S1. (A) Total number of identified lipid species. (B) Relative abundance and (C) species number of each lipid class.**


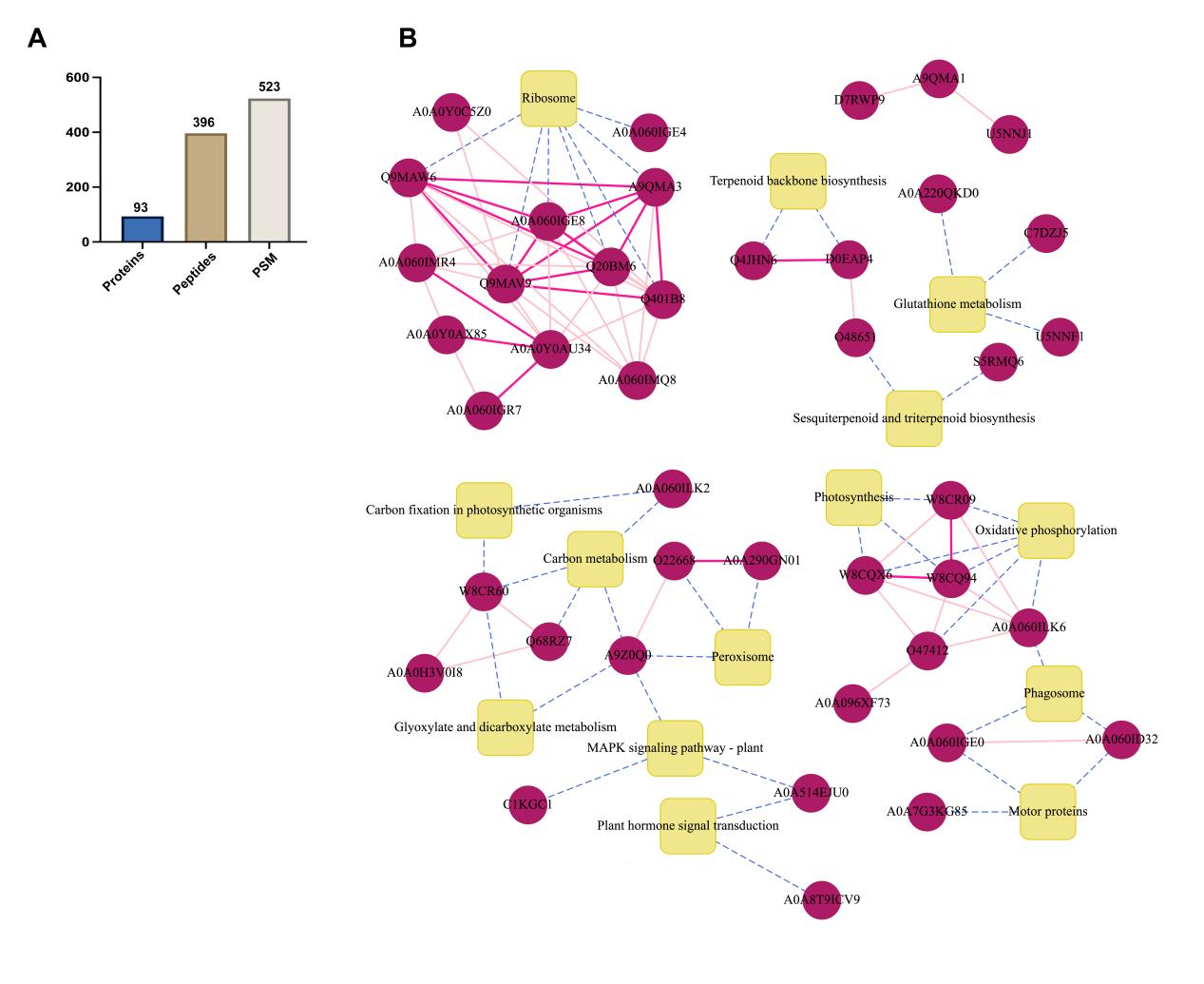


**Figure S2. (A) Statistics of the identification and quantification results, showing the number of spectra, peptides, and proteins. (B) **Protein‒protein** interaction (PPI) network of the identified proteins.**


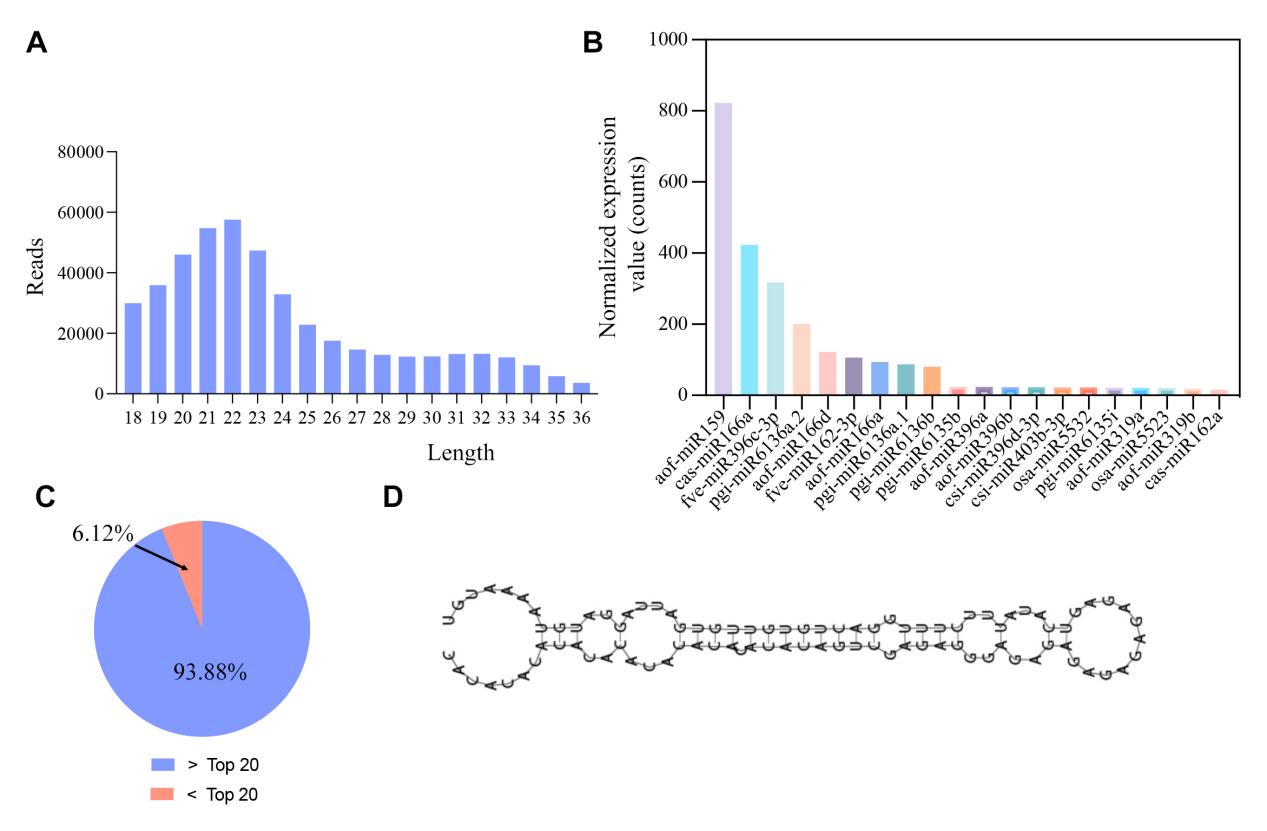


**Figure S3. (A) Length distribution of **the** clean reads. (B) Normalized expression levels of the top 20 most abundant miRNAs. (C) Proportion of the top 20 miRNAs (relative to all miRNAs). (D) Predicted secondary structure plot of a miRNA precursor (representative example).**


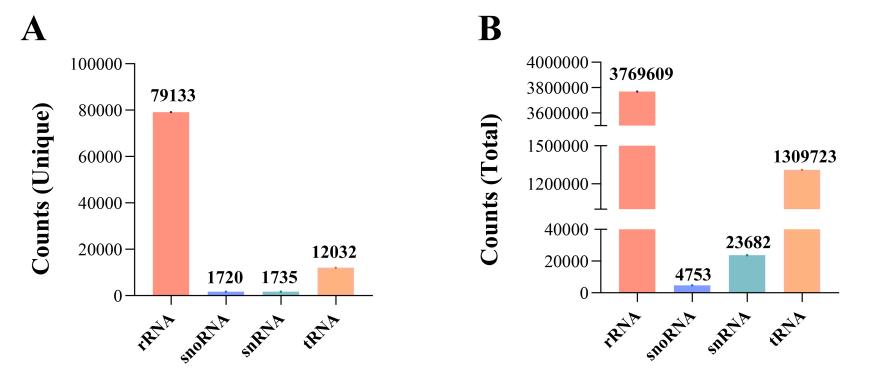


**Figure S4. Classification and abundance of small RNA sequences in G-Exos** **on the **basis of the** Rfam database.**


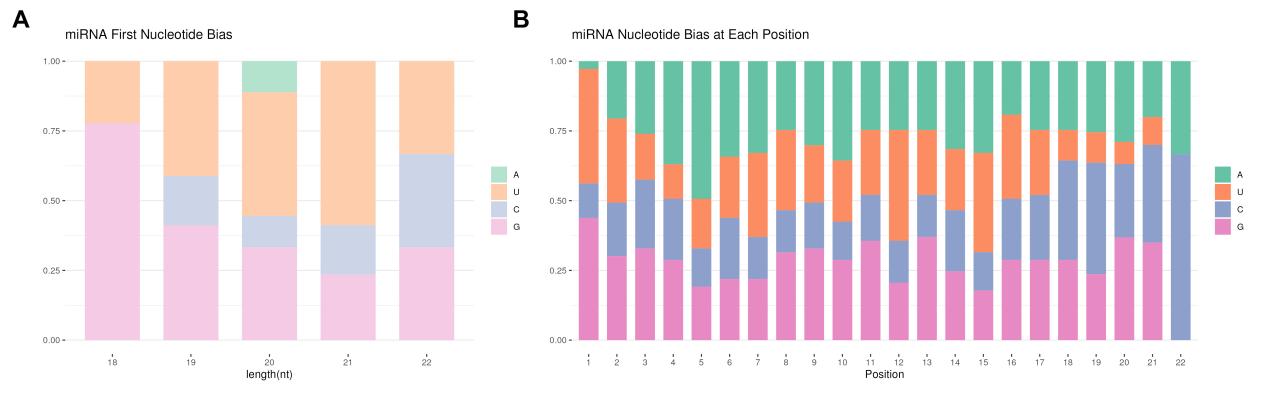


**Figure S5. Nucleotide preference analysis at the 5‘ terminus and each position of mature miRNAs from G-Exos.**


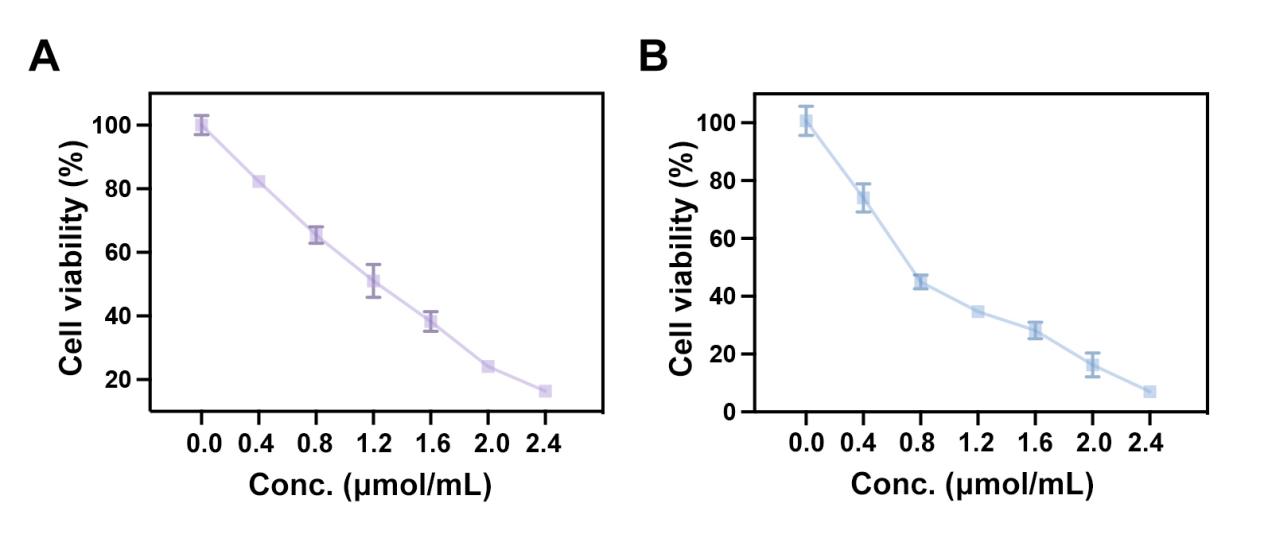


**Figure S6. Viability of (A) HaCaT cells and (B) P1G1 cells after exposure to increasing concentrations of H₂O₂ (0.00 - 2.40 μmol/mL).**


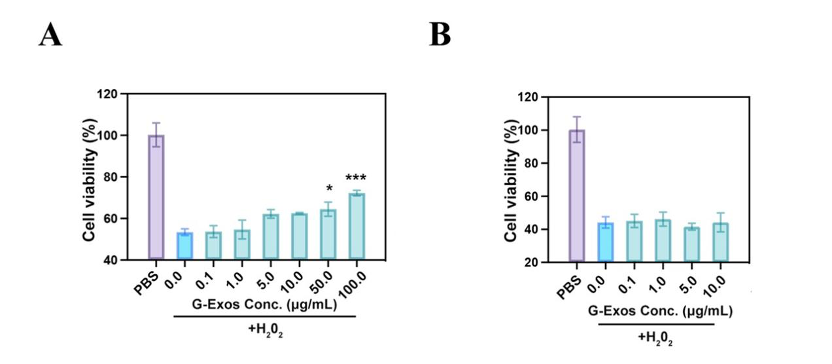


**Figure S7. (A) Viability of HaCaT cells and (B) P1G1 cells after **pre-treatment** with PBS or different concentrations of G-Exos for 24 h followed by exposure to 0.8 μmol/mL H₂O₂.**


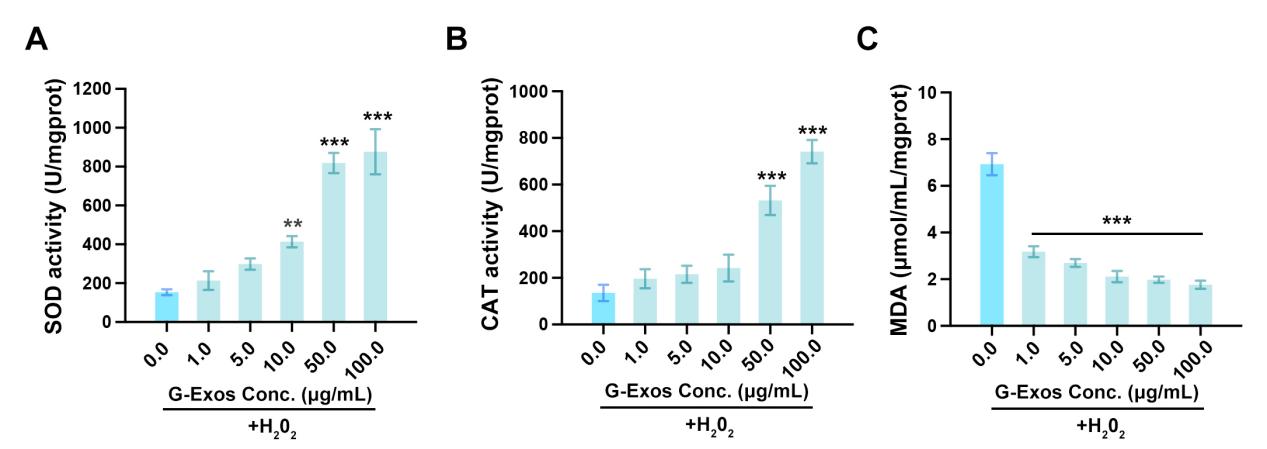


**Figure S8. (A) SOD activity, (B) CAT activity, and (C) MDA content in HACAT cells after exposure to 0.8 μmol/mL H₂O₂ and subsequent treatment with different concentrations of G-Exos for 24 h.**


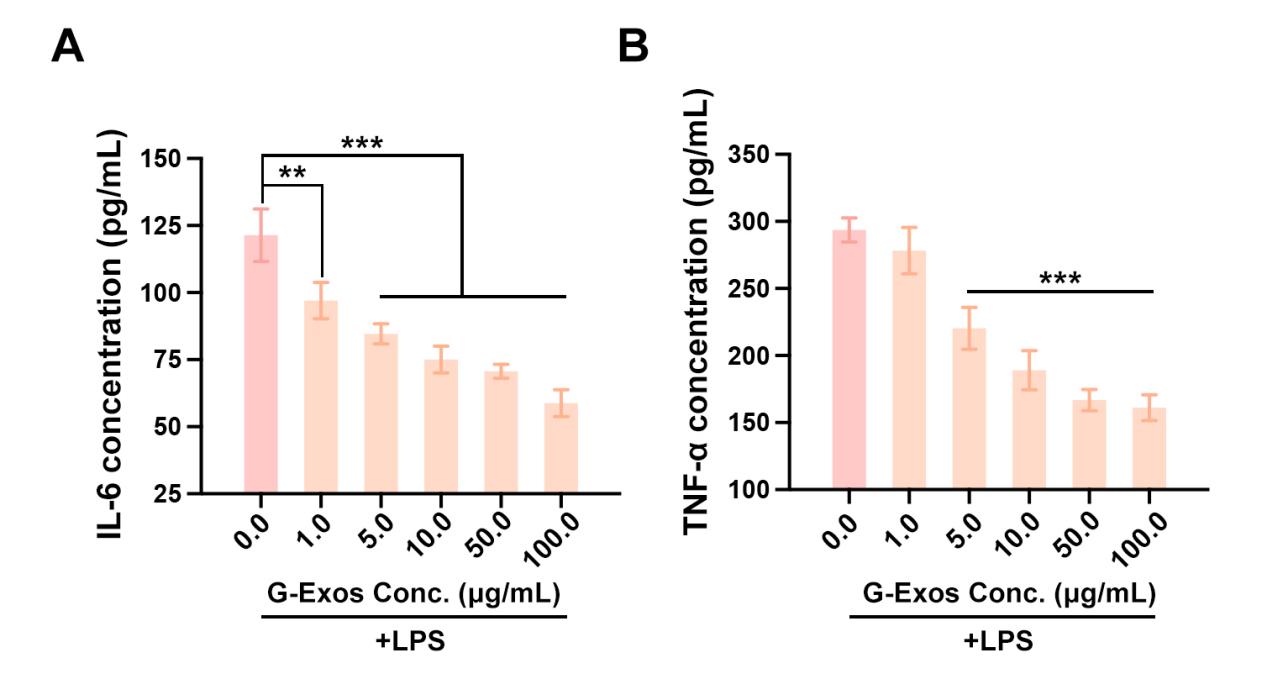


**Figure S9. Concentrations of (A) IL-6 and (B) TNF-α in the HaCaT cell culture supernatant after stimulation with 0.1 ng/mL LPS and subsequent treatment with different concentrations of G-Exos for 24 h.**


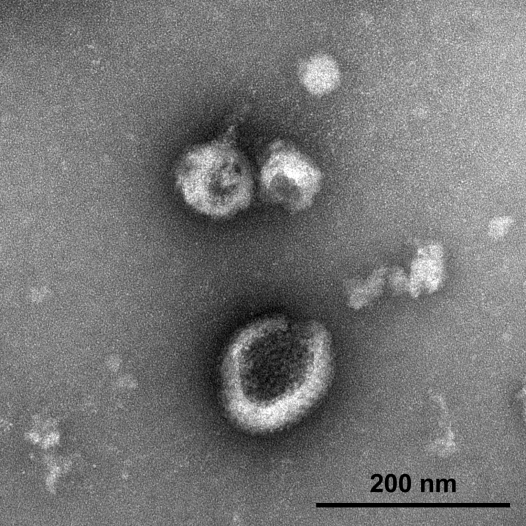


**Figure S10. Representative TEM image of G-Exos released from G-Exos@TM micelles upon MMP-9 stimulation.**


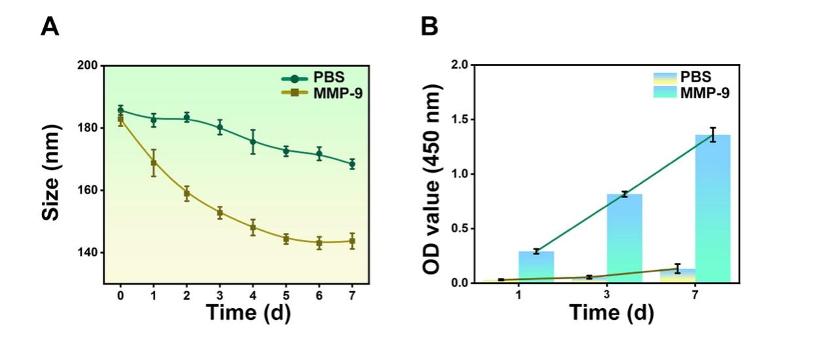


**Figure S11. (A) Size stability of G-Exos@TM in PBS or in the presence of MMP-9 over 7 days. (B) CD63 protein levels of G-Exos@TM after incubation in PBS or MMP-9 for the indicated times.**

**
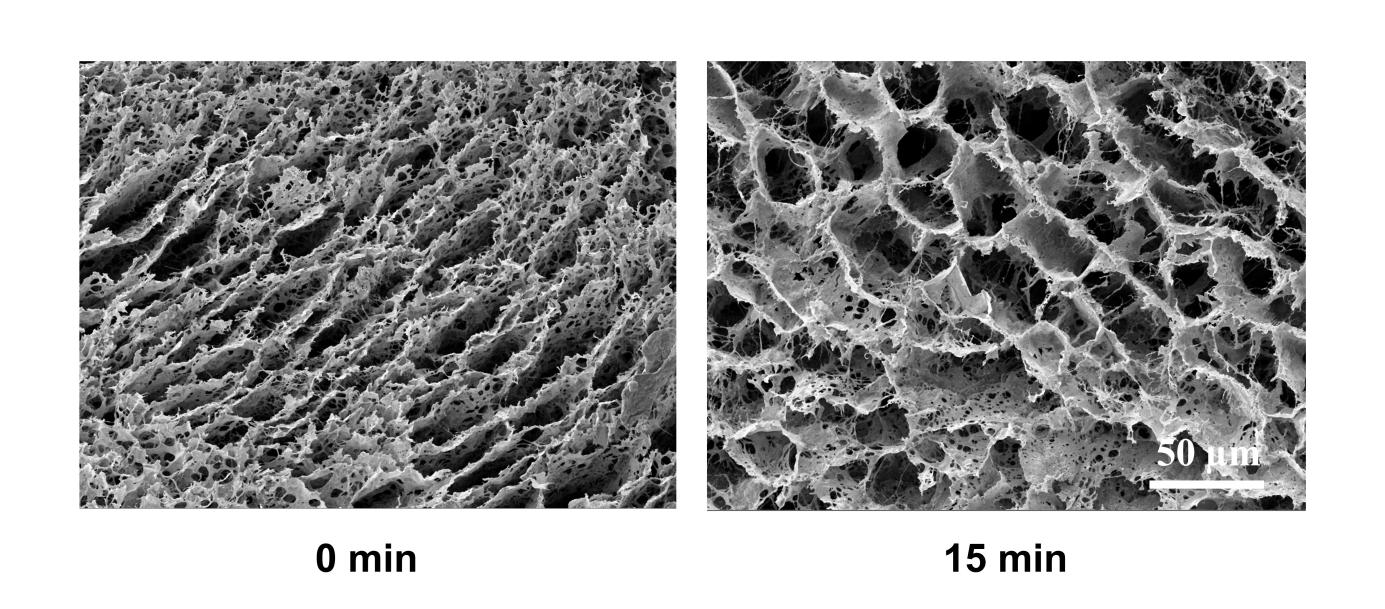
**

**Figure S12. Representative SEM images of microneedles before (0 min) and after (15 min) incubation in PBS.**


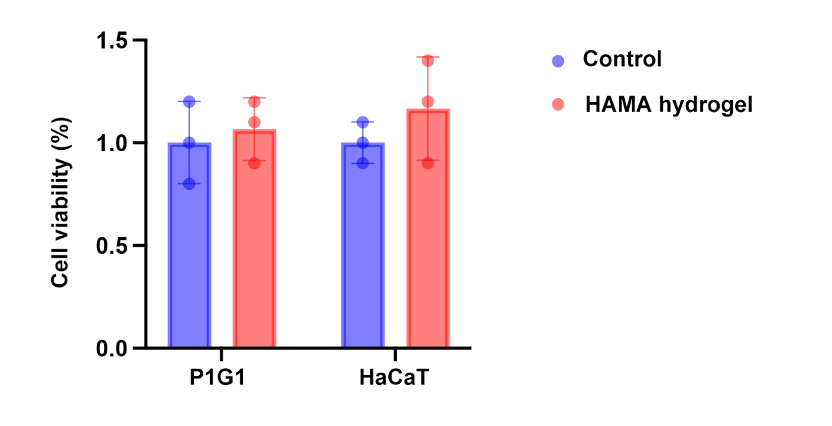


**Figure S13. Representative images showing morphological changes in mouse skin following a single application of HAMA/G-Exos@TM/PDA@PEG MNs.**

**
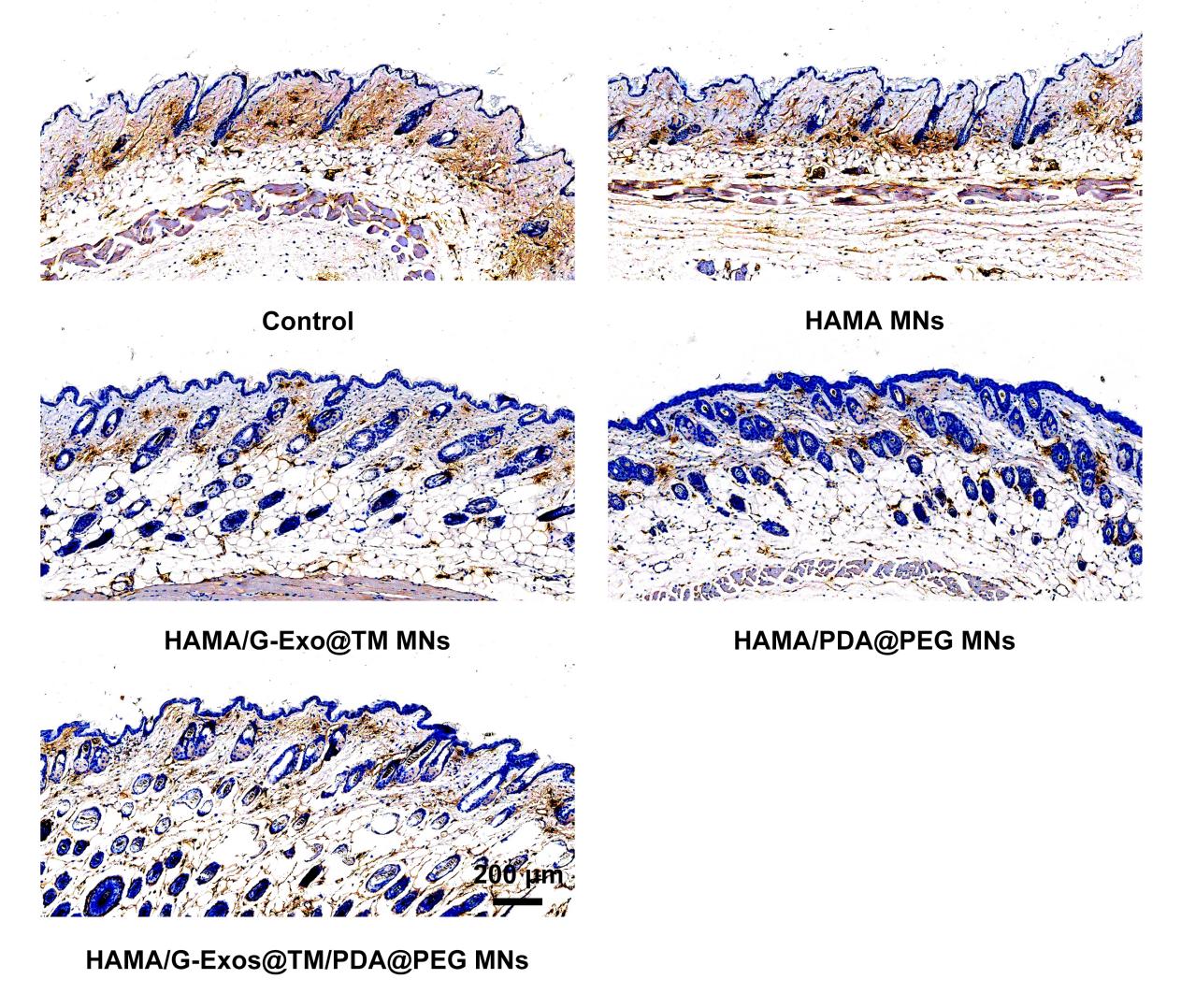
**

**Figure S14. Representative immunohistochemical staining of MMP-9 in skin.**


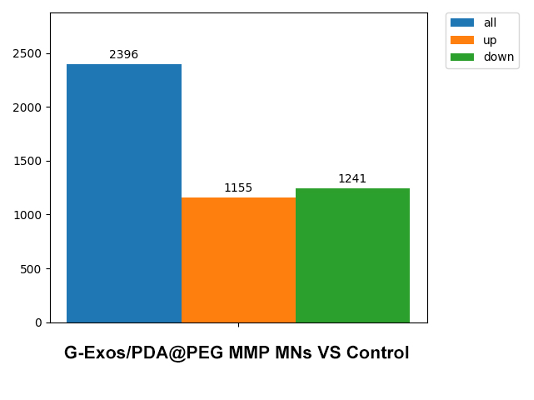


**Figure S15. Statistics of differentially expressed genes (DEGs).**


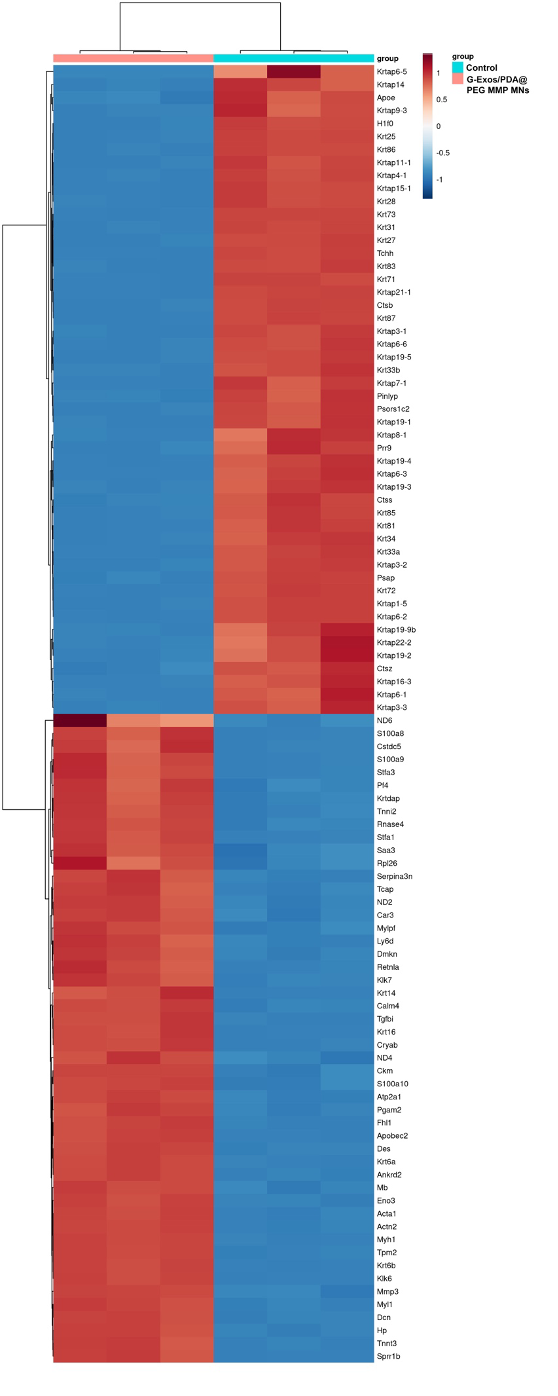


**Figure S16. Clustering analysis of DEGs.**


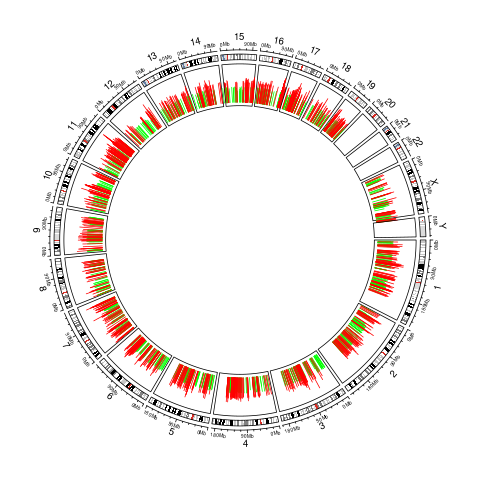


**Figure S17. Chromosomal distribution of DEGs.**


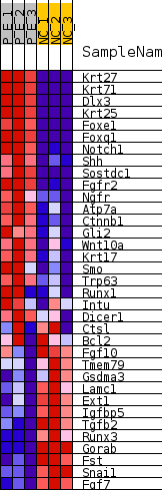


**Figure S18. DEGs involved in hair follicle morphogenesis.**


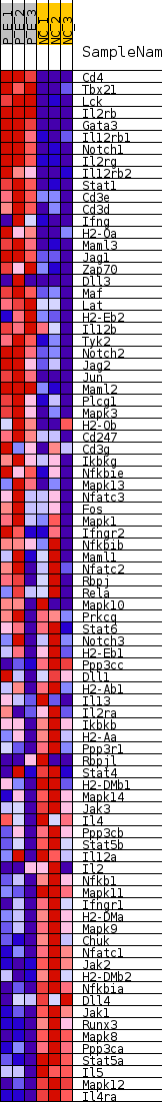


**Figure S19. DEGs involved in Th1 and Th2 cell differentiation.**


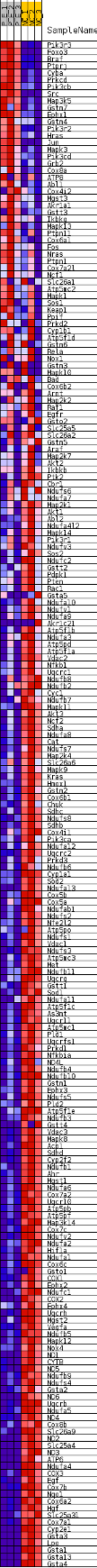


**Figure S20. DEGs involved in Chemical carcinogenesis reactive oxygen specie.**


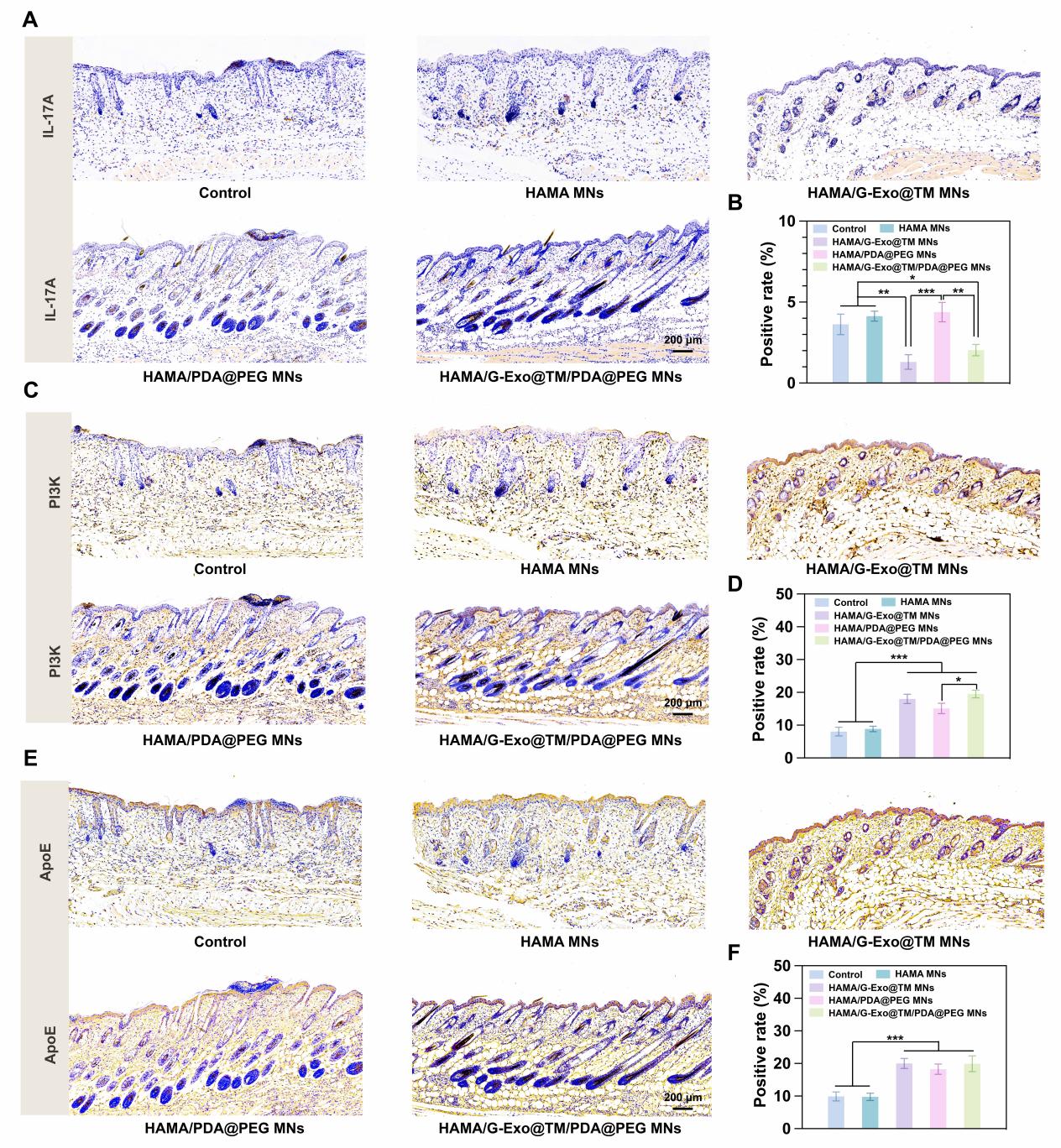


**Figure S21. Representative immunohistochemical staining of IL-17A, PI3K and ApoE in the skin with corresponding quantitative analysis of their expression levels.**

**
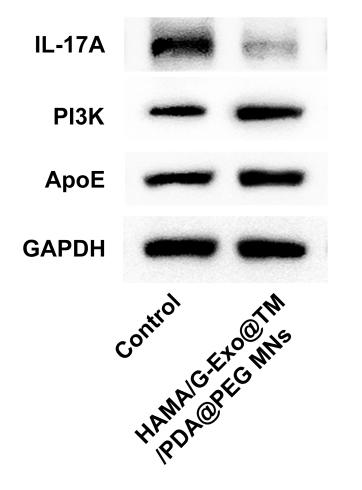
**

**Figure S22. Immunoblots for IL-17A, PI3K, and ApoE in treated skin tissues.**


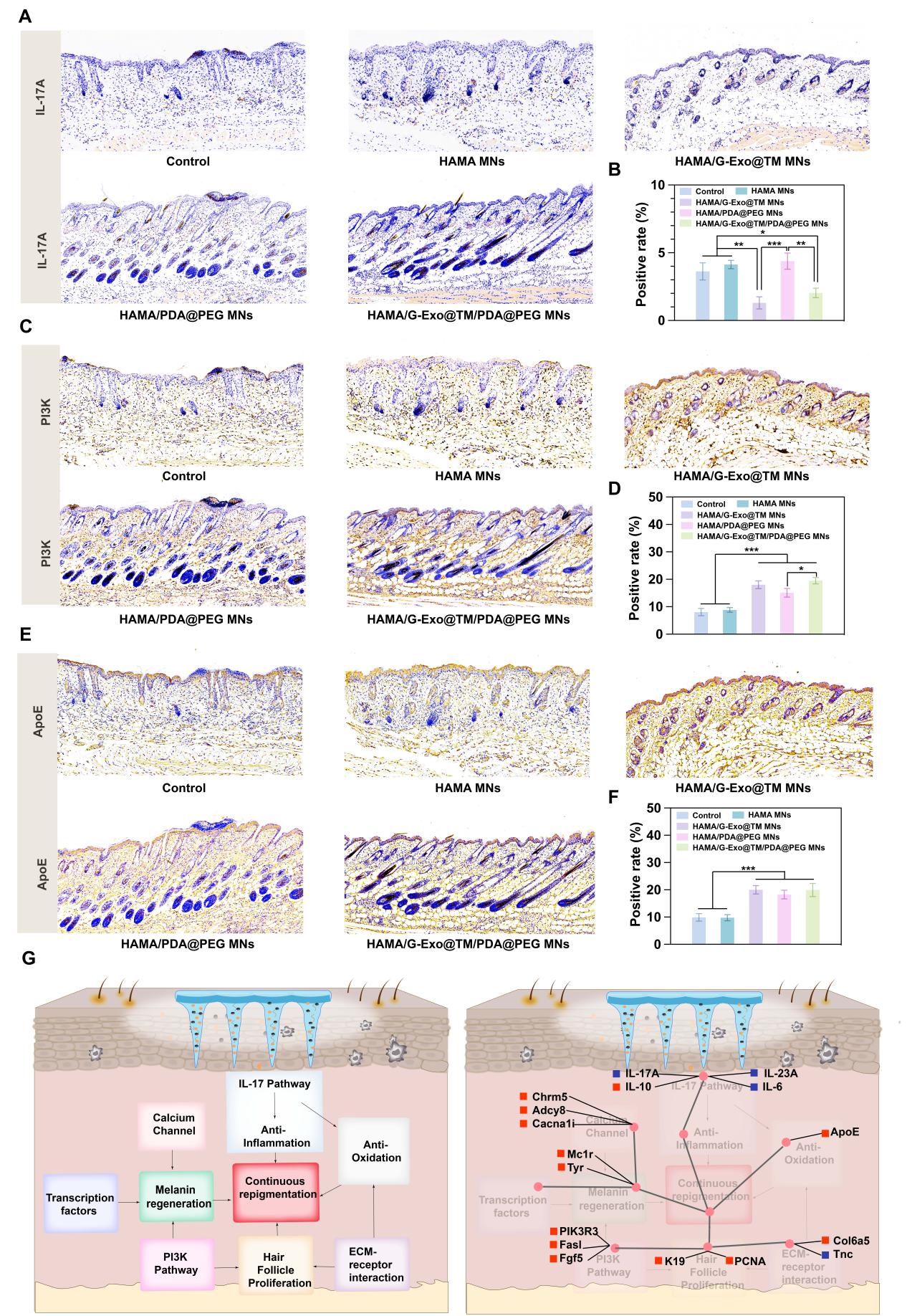


**Figure S23. Schematic diagram illustrating the therapeutic mechanism of the MN system.**

1. Giri, P., D. Desai, and M. Dwivedi, *Animal models unraveling the complexity of vitiligo pathogenesis.* Autoimmun Rev, 2024. **23**(4): p. 103515.

2. Zhu, Y., S. Wang, and A. Xu, *A mouse model of vitiligo induced by monobenzone.* Exp Dermatol, 2013. **22**(7): p. 499-501.
